# The Neuropilin 1 Cytoplasmic Domain Is Required for VEGF-A-Dependent Arteriogenesis

**DOI:** 10.1016/j.devcel.2013.03.019

**Published:** 2013-04-29

**Authors:** Anthony Lanahan, Xi Zhang, Alessandro Fantin, Zhen Zhuang, Felix Rivera-Molina, Katherine Speichinger, Claudia Prahst, Jiasheng Zhang, Yingdi Wang, George Davis, Derek Toomre, Christiana Ruhrberg, Michael Simons

**Affiliations:** 1 Yale Cardiovascular Research Center, Section of Cardiovascular Medicine, Department of Internal Medicine, Yale University School of Medicine, New Haven, CT 06511, USA; 2 Department of Cell Biology, Yale University School of Medicine, New Haven, CT 06511, USA; 3 Institute of Ophthalmology, University College London, London EC1V 9EL, UK; 4 Department of Medical Pharmacology and Physiology, University of Missouri School of Medicine, Columbia, MO 65212, USA

## Abstract

Neuropilin 1 (NRP1) plays an important but ill-defined role in VEGF-A signaling and vascular morphogenesis. We show that mice with a knockin mutation that ablates the NRP1 cytoplasmic tail (*Nrp1*^*cyto*^) have normal angiogenesis but impaired developmental and adult arteriogenesis. The arteriogenic defect was traced to the absence of a PDZ-dependent interaction between NRP1 and VEGF receptor 2 (VEGFR2) complex and synectin, which delayed trafficking of endocytosed VEGFR2 from Rab5+ to EAA1+ endosomes. This led to increased PTPN1 (PTP1b)-mediated dephosphorylation of VEGFR2 at Y^1175^, the site involved in activating ERK signaling. The *Nrp1*^*cyto*^ mutation also impaired endothelial tubulogenesis in vitro, which could be rescued by expressing full-length NRP1 or constitutively active ERK. These results demonstrate that the NRP1 cytoplasmic domain promotes VEGFR2 trafficking in a PDZ-dependent manner to regulate arteriogenic ERK signaling and establish a role for NRP1 in VEGF-A signaling during vascular morphogenesis.

## Highlights

• The NRP1 cytoplasmic domain promotes VEGF receptor (VEGFR) 2 endocytic trafficking
• In its absence, VEGR2 trafficking is delayed in sorting endosomes
• PTP1b binds to Rab5+ sorting endosomes and dephosphorylates the Y^1175^ site of VEGFR2
• Loss of the NRP1 cytoplasmic domain impairs developmental and adult arteriogenesis

## Graphical Abstract

**Figure:**
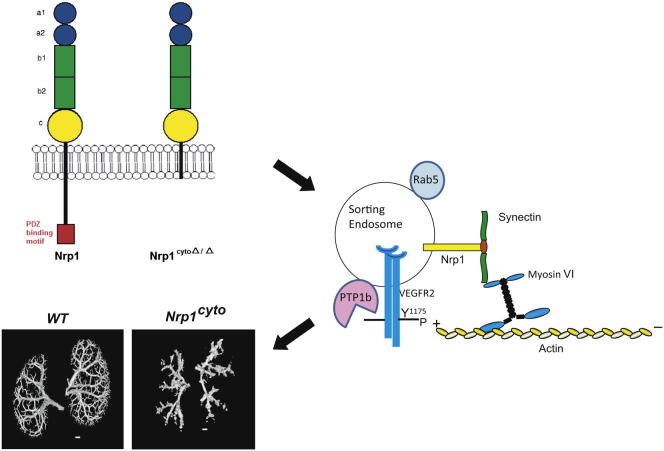

## Teaser

Lanahan et al. find that the VEGF coreceptor NRP1 is required for proper VEGFR2 endocytic trafficking. In knockin mice lacking NRP1’s cytoplasmic domain, VEGFR2 passes through early endocytic compartments more slowly, increasing its exposure to the inhibitory phosphatase PTP1b and attenuating VEGF-stimulated ERK signaling and arteriogenesis.

## Introduction

Vascular morphogenesis requires the three complementary processes of vasculogenesis, angiogenesis, and arteriogenesis. All three are VEGF-A dependent, but how VEGF-A signaling is regulated at the molecular level to selectively stimulate each process at the appropriate time is poorly understood (Carmeliet and Jain, 2011). VEGF-A exerts its biological effects by binding to its tyrosine kinase (TK) receptors VEGFR1 and VEGFR2 and the non-TK receptor neuropilin 1 (NRP1) (Koch et al., 2011). In endothelial cells, VEGFR1 is thought to function as a “decoy” receptor, whereas VEGFR2 is the main signal transducer of all major biological effects, including endothelial cell proliferation, migration, and survival. NRP1 was first identified as a semaphorin 3A receptor, but can also bind the VEGF-A_165_ isoform of VEGF-A with high affinity (Koch, 2012). Although NRP1’s precise role in VEGF-A signaling in vivo has not been defined, its extracellular domain is thought to act in a manner similar to other coreceptors, such as syndecans and glypicans, by increasing the local plasma membrane concentration of VEGF-A and thereby promoting its binding to VEGFR2 (Koch, 2012; Koch et al., 2011; Sorkin and von Zastrow, 2009).

NRP1 possesses a short cytoplasmic domain that contains a PDZ-binding domain at its C terminus (Wang et al., 2006). Yeast-two hybrid studies identified the PDZ-domain protein synectin (also known as neuropilin 1-binding protein NIP or GIPC1) as a binding partner for the NRP1 cytoplasmic tail (Cai and Reed, 1999), and this interaction has been confirmed in endothelial cells (Prahst et al., 2008). Synectin is an essential regulator of arterial morphogenesis that acts by promoting VEGFR2 endocytosis and signaling (Chittenden et al., 2006; Lanahan et al., 2010). However, it remains unknown how VEGFR2 interacts with synectin, as it lacks a PDZ binding domain. One possibility is that the adaptor protein APPL1 couples early endosomes containing VEGFR2 to the cytoplasmic trafficking machinery, as it has a PDZ-domain (Lin et al., 2006). Another possibility is that NRP1 provides this link, as it has the ability to bind both VEGF-A_165_ and synectin (Cai and Reed, 1999). To identify if NRP1 plays a specific role in arteriogenesis, and, in particular, to examine if the NRP1-synectin interaction promotes VEGF-A-induced VEGFR2 signaling during arteriogenesis, we studied *Nrp1*^*cyto*^ mice that lack the exon that encodes the NRP1 cytoplasmic domain.

*Nrp1*^*cyto*^ mice are viable and display normal developmental angiogenesis, with the only abnormality reported to date being an increased frequency of artery-vein crossings in the retina (Fantin et al., 2011). We show here that pathological angiogenesis is also normal in these mice, whereas, arterial morphogenesis, during development and in adults, was significantly impaired. The observed abnormalities included a reduced number and branching frequency of small arteries in various organs and an impaired arteriogenic response after arterial injury. Furthermore, endothelial cells from *Nrp1*^*cyto*^ mice showed impaired ERK activation in response to VEGF-A_165_ and inefficiently assembled endothelial tubules in vitro. We traced these defects to reduced VEGF-A_165_-induced trafficking of VEGFR2, which resulted in its prolonged exposure to the protein tyrosine phosphatase (PTP) 1b in Rab5+ endosomes and therefore decreased phosphorylation of the VEGFR2 tyrosine Y1175 that activates ERK signaling. Abnormal ERK signaling in *Nrp1*^*cyto*^ endothelial cells could be rescued by full-length NRP1, but not NRP1 lacking the cytoplasmic domain or its PDZ-binding sequence. Furthermore, abnormal endothelial cell (EC) tubulogenesis in vitro could be rescued by expression of full-length NRP1 or constitutively active ERK. These findings establish NRP1 as a specific regulator of VEGF-A-induced VEGFR2 trafficking and ERK signaling that is essential for VEGF-A-dependent arterial morphogenesis.

## Results

To evaluate the role of the NRP1 cytoplasmic domain in postnatal angiogenesis and arteriogenesis, we studied *Nrp1*^*cyto*^ knockin mice, in which a stop codon in exon 17 prevents translation of the cytoplasmic domain (Fantin et al., 2011). We first studied the angiogenic response of these mice in several different models of postnatal and pathological vessel growth and then concentrated on developmental and postnatal arteriogenesis.

### Unaltered Pathological Angiogenesis in *Nrp1*^*cyto*^ Mice

We have previously shown that the NRP1 cytoplasmic tail is not essential for normal angiogenesis in the developing mouse retina (Fantin et al., 2011), a finding confirmed in the present study (Figures S1A and S1A′ available online). To address if this NRP1 domain is instead required for pathological angiogenesis in this tissue, we employed a mouse model of oxygen-induced retinopathy (Smith et al., 1994). In this assay, 7-day-old mouse pups with a partially developed retinal vasculature are exposed to hyperoxia for 5 days. This treatment obliterates immature capillaries in the central retina, in particular around arteries with their high oxygen tension, but does not cause regression of arteries or veins (Figure S1B, “vo” indicates an area with vasoobliteration). After return to normoxic room air on day 12, the now poorly vascularized retina becomes hypoxic, upregulates VEGF-A, and initiates a pathological, neovascular response (Figure S1B, “nv” indicates an area with neovascularization). Specifically, the neovascular response leads to the longitudinal expansion of arteries, leading to their tortuous appearance (Figures S1B and S1B′, arrowhead). At the same time, sprouting from veins and remaining capillaries leads to the formation of neovascular tufts (Figure S1B′, straight and curved arrows, respectively). The pattern of vasoobliteration and neovascularization appeared similar in *Nrp1*^*cyto*^ mice and their wild-type (WT) littermates (Figures S1B, S1C, S1B′, and S1C′). Quantitation confirmed that there was no significant difference in vasoobliteration or the neovascular response in both genotypes (Figures S1D and S1E).

We next studied angiogenesis in subcutaneous implants of growth factor-depleted Matrigel pellets supplemented with buffer (controls) or the NRP1-binding isoform of VEGF-A, VEGF-A_165_. VEGF-A_165_-loaded pellets induced an equally strong angiogenic response in control and *Nrp1*^*cyto*^ mice after 7 days (Figures S1F and S1G). Finally, we used a skin wounding model, in which wound closure is strongly dependent on angiogenesis (Tonnesen et al., 2000). We found that the rate of wound closure was similar in control and *Nrp1*^*cyto*^ mice over a course of 7 days (Figures S1H and S1I). Taken together, all three assays show that the NRP1 cytoplasmic tail is not required for postnatal or pathological angiogenesis.

### Impaired Arteriogenesis in *Nrp1*^*cyto*^ Mice

**Figure 1:**
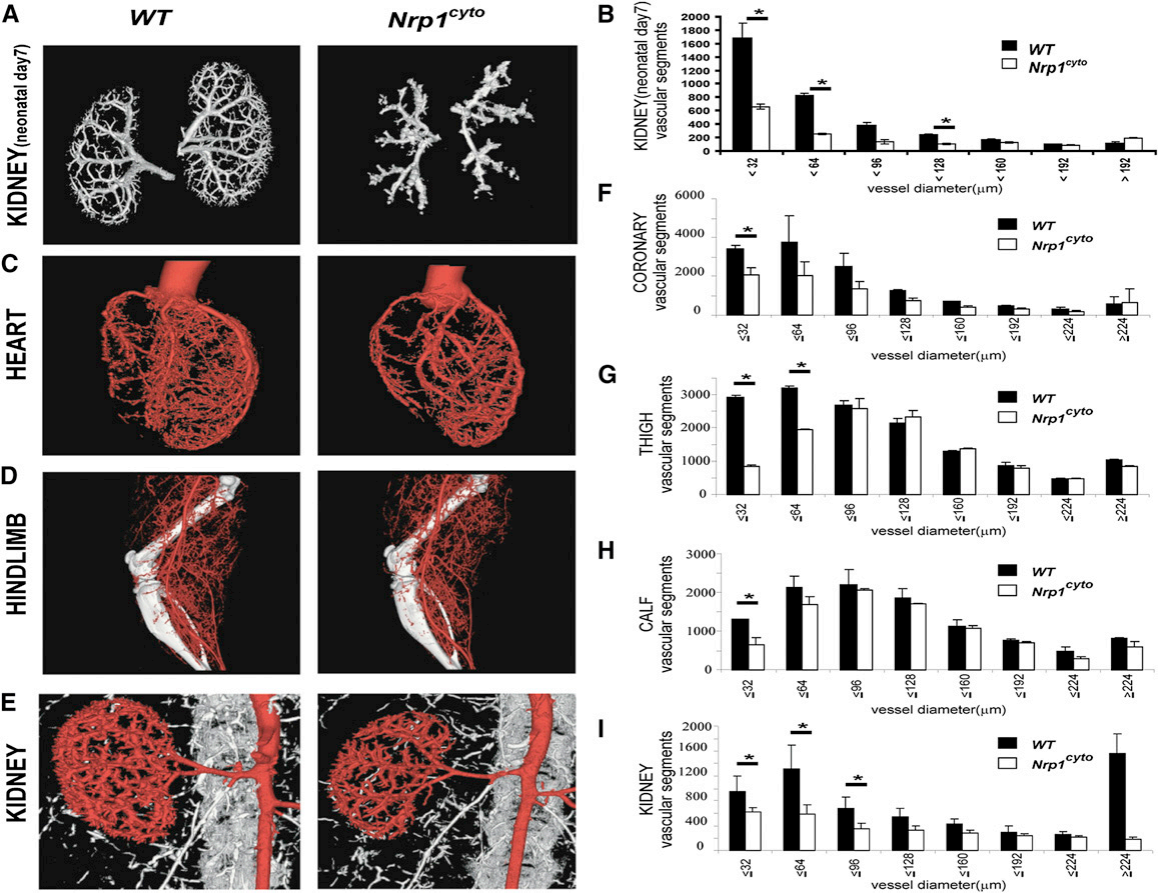
Micro-CT Analysis of the Arterial Vasculature Kidney of Neonatal Day 7 Kidney and in Heart, Hindlimb, and Kidney of Adult *Nrp1*^*cyto*^ Mice and Wild-Type Littermates (A–D) Representative reconstructed micro-CT images of day 7 neonatal kidney and the heart, hindlimb, and kidney of WT and *Nrp1*^*cyto*^ mice at 16 μm resolution. (E–I) Quantitative analysis of micro-CT images from *Nrp1*^*cyto*^ (white bars) and WT (black bars) neonatal kidney and adult heart, hindlimb, and kidney shows a significant decrease in the total number of <100 μm diameter vessels (mean ± SEM, ^*^p < 0.05). See also Figures S1 and S2.

We then examined the requirement of the NRP1 cytoplasmic tail for developmental and adult arteriogenesis. To this end, we used micro-CT (mCT) to visualize arterial circulation in the kidneys of neonatal (P7) and in the heart, hindlimbs, and kidneys of young adult (6–8 weeks of age) mice, using contrast injections that fill only arterial, but not capillary or venous beds (Simons, 2008). The mCT images of the arterial circulation and their quantitative analysis showed a significant reduction in the number of smaller arteries and arterioles in *Nrp1*^*cyto*^ mice compared to littermate controls in developing renal vasculature at P7 (Figures 1A and 1B) and in the adult coronary, limb and renal circulations (Figures 1C–1I). Overall, the pattern of the arterial tree in *Nrp1*^*cyto*^ mice closely resembled that of synectin null mice (Chittenden et al., 2006). Consistent with an impaired development of the arterial vascular system in different organs, adult *Nrp1*^*cyto*^ mice showed a small, but significant reduction in body, kidney, and heart weight (Figure S2A) as well as mCT-determined kidney and heart size (Figure S2B) compared to WT littermates.

**Figure 2:**
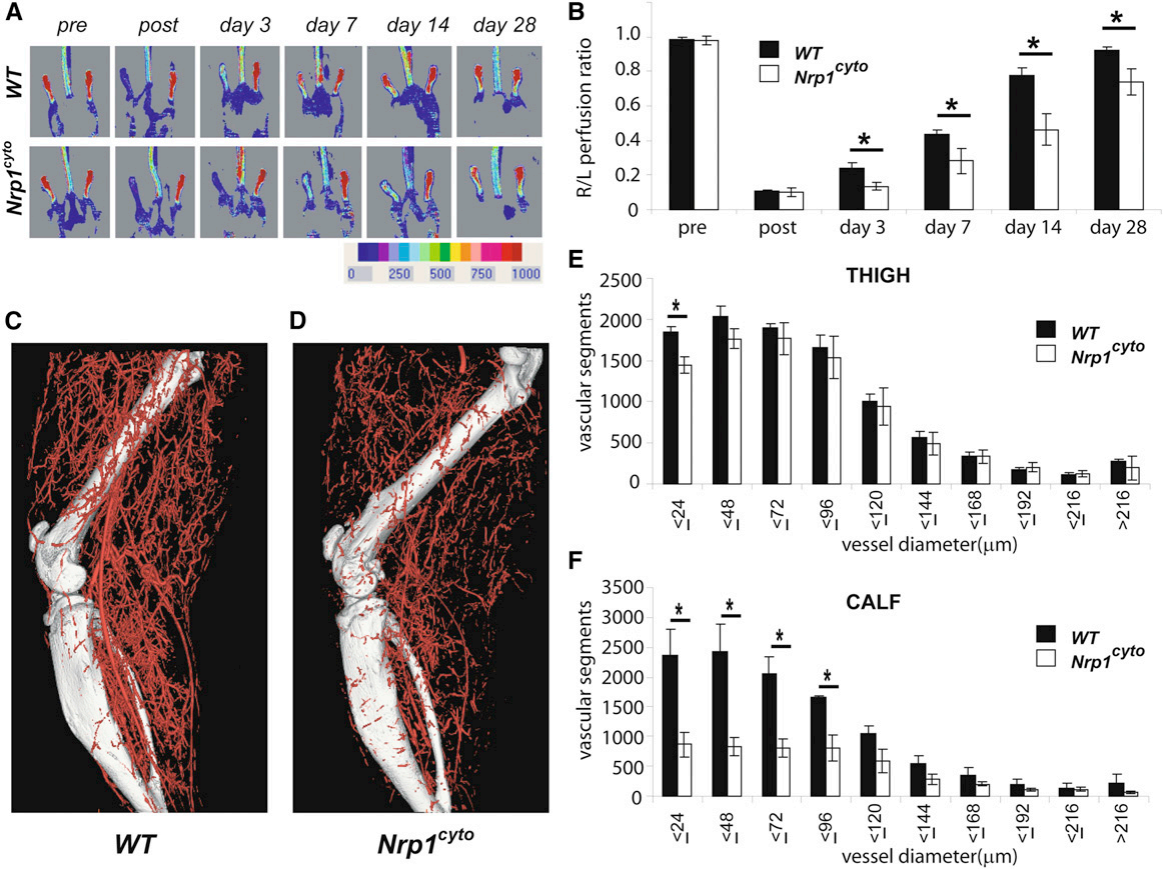
*Nrp1*^*cyto*^ Mice Show Impaired Blood Flow Recovery in the Hindlimb Ischemia Model (A and B) Blood flow measurements in the HLI model. (A) Doppler imaging demonstrated the level of perfusion in hindlimbs of WT and *Nrp1*^*cyto*^ mice before and after HLI surgery at the indicated time points. (B) Quantitative analysis of laser Doppler images 3 days after HLI surgery in WT (black bars) and in *Nrp1*^*cyto*^ mice (mean ± SEM, ^*^p < 0.05). (C–F) Micro-CT analysis of arterial diameter in the HLI model. Representative micro-CT images of (C) WT and (D) *Nrp1*^*cyto*^ mice 14 days after HLI surgery. Quantitative micro-CT analysis of arterial vasculature (E) above and (F) below the knee in WT (black bars) and *Nrp1*^*cyto*^ mice (white bars) 14 days after HLI surgery. The total number of <100 μm diameter vessels was significantly decreased in *Nrp1*^*cyto*^ mice relative to WT littermates in both the thigh and calf (mean ± SEM, ^*^p < 0.05). See also Figure S3.

We next analyzed arteriogenesis in the hindlimb ischemia model, which was induced by the ligation and removal of a segment of the common femoral artery, as previously described (Ren et al., 2010). Blood flow measurements with a deep penetrating laser-Doppler probe demonstrated comparable blood flow before injury and a 90% reduction in perfusion in both *Nrp1*^*cyto*^ mutants and WT littermates immediately after induction of ischemia (Figures 2A and 2B). Although flow recovered over a period of 2 weeks in control mice, flow recovery in *Nrp1*^*cyto*^ mice was reduced as early as day 3, remained at ∼50% of control levels at day 14, and had not fully recovered by day 28 (Figures 2A and 2B).

Because arteriogenesis is the key process responsible for blood flow recovery in this model (Chittenden et al., 2006), we used mCT to visualize and quantitate the extent of the arterial circulation (Figures 2C and 2D). We observed a significant decrease in the density and number of smaller arteries and arterioles both above and below the knee in *Nrp1*^*cyto*^ compared to control mice (Figures 2E and 2F). This difference could not be explained by low synectin levels, which we have previously shown to impair arteriogenesis in this experimental setting (Chittenden et al., 2006) as western blot analysis of normal and ischemic tissues from both WT and *Nrp1*^*cyto*^ mice demonstrated similar synectin expression (Figure S3A).

### Impaired VEGF-A Signaling and ERK Activation in *Nrp1*^*cyto*^ Mice

**Figure 3:**
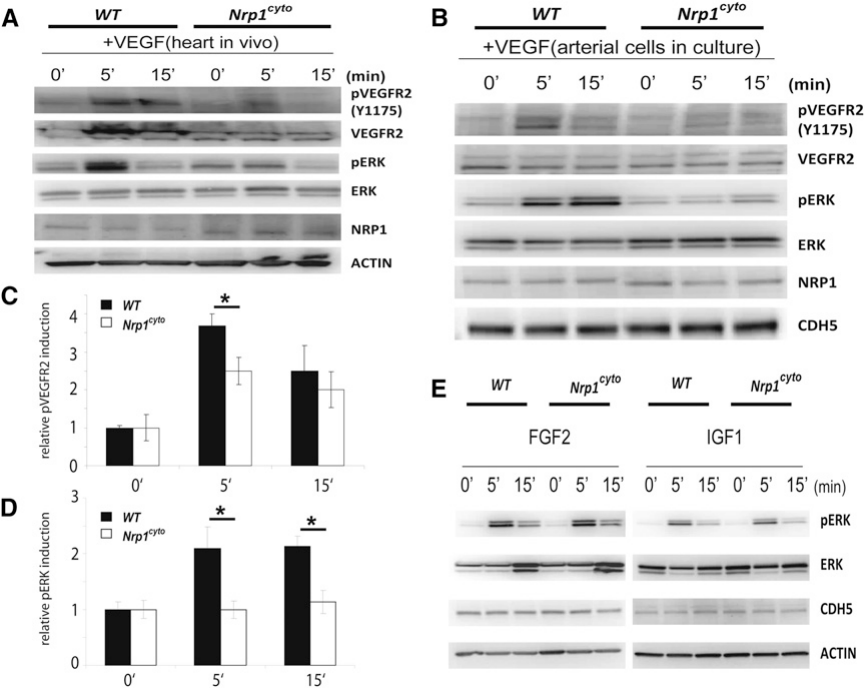
Impaired VEGF-A Signaling in *Nrp1*^*cyto*^ Mice (A) Western blot analysis of heart lysates following intraperitoneal injection of VEGF-A. Blots show reduced ERK and VEGFR2 phosphorylation in *Nrp1*^*cyto*^ mice relative to WT littermates. (B–D) Western blot analysis of cell lysates from primary arterial EC from *Nrp1*^*cyto*^ and WT EC that were serum-starved and then stimulated for the indicated times with 50 ng/ml VEGF-A_165_. (B) Blots show reduced phosphorylation of VEGFR2 on Y1175 and of ERK in *Nrp1*^*cyto*^ relative to control EC. (C) Quantification of the ratio of phospho-VEGFR2 (pVEGFR2) relative to total VEGFR2 (VEGFR2) (mean ± SD, n = 5, ^*^p < 0.05). (D) Quantification of the ratio of phospho-ERK (pERK) relative to total ERK (ERK) (mean ± SD, n = 5, ^*^p < 0.05). (E) Western blot analysis of cell lysates from *Nrp1*^*cyto*^ and WT primary arterial EC that were serum-starved and stimulated for the indicated times with 50 ng/ml of FGF2 or IGF1. ERK phosphorylation was similar in *Nrp1*^*cyto*^ and WT EC. See also Figure S4.

Impaired arteriogenesis has been linked to decreased activation of ERK signaling by VEGF-A (Hong et al., 2006; Lanahan et al., 2010). Indeed, immunofluorescence analysis of forming collaterals in the hindlimb ischemia model demonstrated reduced ERK activation in the endothelium of *Nrp1*^*cyto*^ compared to WT mice (Figure S3B, white arrows). To analyze the contribution of the NRP1 cytoplasmic tail to this process, we first compared VEGF-A_165_-induced ERK activation in *Nrp1*^*cyto*^ and control mice in vivo. Tissue lysates from control and *Nrp1*^*cyto*^ hearts demonstrated a slight increase in Nrp1 levels in the mutant mice consistent with prior observations (Fantin et al., 2011). Injection of 1 μg/kg VEGF-A_165_ intraperitoneally (i.p.) led to a rapid upregulation of VEGFR2 phosphorylation at the Y^1175^ residue that is involved in the activation of PLCγ/ERK signaling in control mice and was accompanied by increased ERK1/2 phosphorylation (Figure 3A). Strikingly, both of these responses to VEGF-A_165_ were markedly reduced in *Nrp1*^*cyto*^ hearts (Figure 3A).

To obtain further information on the molecular mechanism involved in reduced ERK activation after loss of the NRP1 cytoplasmic tail, we next examined VEGF-A signaling in primary arterial EC from *Nrp1*^*cyto*^ and control mice. As in vivo, NRP1 levels were slightly elevated in *Nrp1*^*cyto*^ EC compared to WT EC, whereas VEGFR2 expression was similar in EC from both genotypes (Figure 3B). Compared to WT EC, *Nrp1*^*cyto*^ EC showed a marked decrease in phosphorylation of the VEGFR2 Y1175 site (Figures 3B and 3C). In agreement with this observation and our in vivo findings, ERK1/2 phosphorylation was also significantly decreased in the mutant primary EC (Figures 3B and 3D).

To investigate whether the reduced ERK activation in *Nrp1*^*cyto*^ EC is specific to impaired VEGF-A signaling, or reflects a broader signaling defect, we examined the effect of both FGF2 and IGF1 treatment on ERK signaling in primary EC from *Nrp1*^*cyto*^ and control mice. Both growth factors activated ERK similarly in EC from both genotypes (Figure 3E). These results demonstrate that impaired ERK1/2 signaling in EC of *Nrp1*^*cyto*^ mice is specific to VEGF-A_165_-induced VEGFR2 activation.

To exclude that impaired VEGFR2 phosphorylation simply reflects a reduced affinity of NRP1^cyto^ for VEGF-A_165_, we used an established method that tests binding of NRP1 ligands such as alkaline phosphatase-conjugated VEGF-A_165_ to NRP1-expressing axons and vessels in vivo (Vieira et al., 2007). Using this assay, we observed similar binding of VEGF-A_165_ to axons that express NRP1, but lack VEGFR2, and to vessels that coexpress NRP1 and VEGFR2 in *Nrp1*^*cyto*^ mice and controls (Figure S4A). In contrast, VEGF-A_165_ failed to bind to axons that lack VEGFR2 in the *Nrp1*^*−/−*^ brain, as expected (Figure S4B). These results agree with previous in vitro studies, which used Scatchard analysis to show a similar affinity of VEGF-A_165_ for full-length NRP1 and NRP1 lacking the cytoplasmic PDZ binding domain (Prahst et al., 2008).

**Figure 4:**
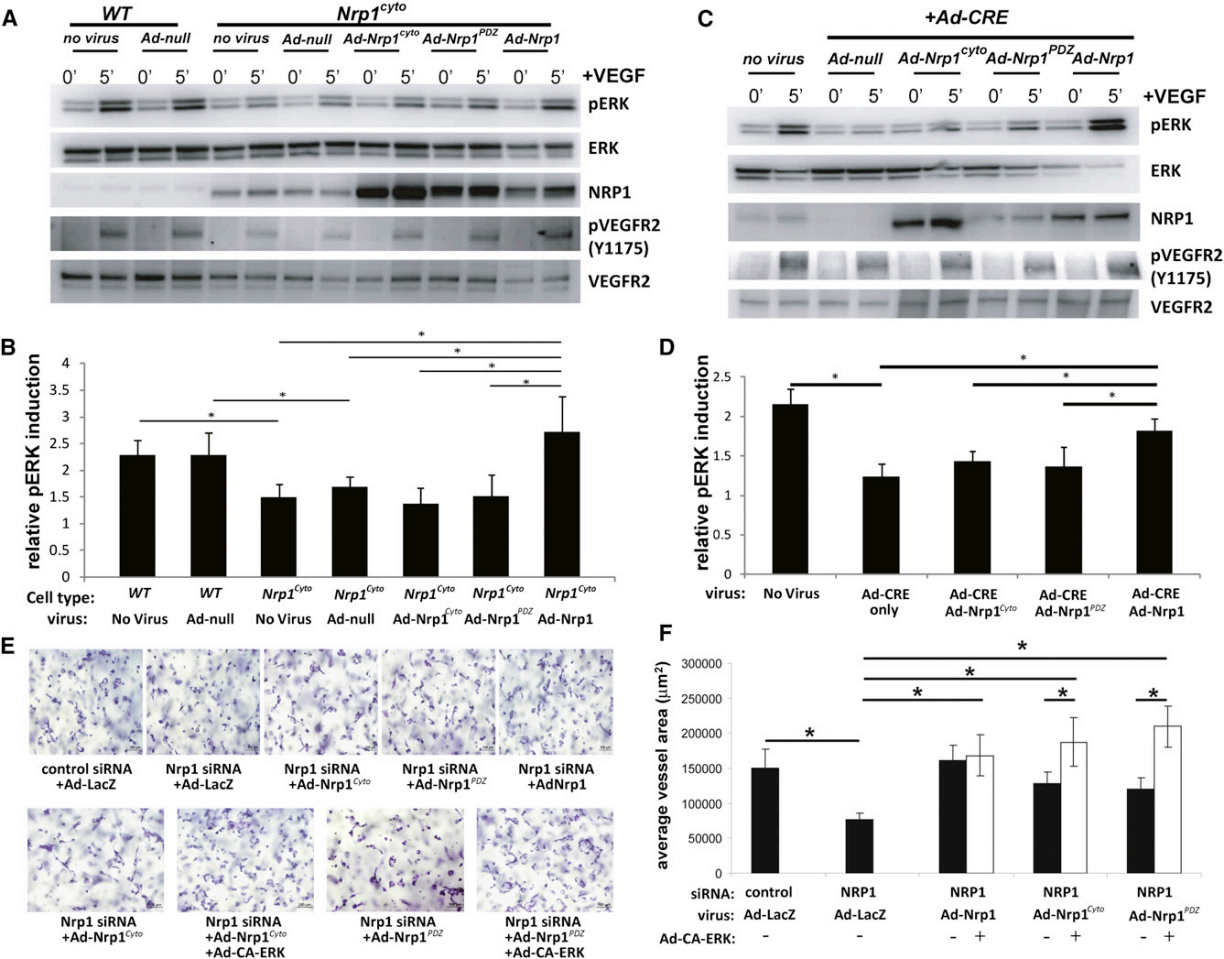
Rescue of VEGF-A Signaling in *Nrp1*^*cyto*^ and *Nrp1*^*fl/fl*^ Primary EC by Full-Length NRP1 and of EC Tubulogenesis following NRP1 siRNA Knockdown with Constitutively Active ERK (A and B) Western blot analysis of *Nrp1*^*cyto*^ EC that were transduced with the indicated adenoviral constructs, serum-starved and stimulated with 50 ng/ml VEGF-A_165_. Blots (A) and quantitation of ERK activation (B) show that *Ad-Nrp1*, but not *Ad-Nrp1*^*cyto*^ or *Ad-Nrp1*^*PDZ*^, restores VEGF-A-induced VEGFR2 and ERK activation in *Nrp1*^*cyto*^ EC (mean ± SD, n = 3, ^*^p < 0.05). (C and D) Western blot analysis of *Nrp1*^*fl/fl*^ EC treated with adenoviral constructs expressing CRE recombinase to inactivate NRP1 were transduced with the indicated adenoviral constructs, serum-starved and stimulated with 50 ng/ml VEGF-A_165_. Blots (C) and quantitation (D) show that *Ad-Nrp1*, but not *Ad-Nrp1*^*cyto*^ or *Ad-Nrp1*^*PDZ*^, restores the activation of VEGFR2 and ERK (mean ± SD, n = 3, ^*^p < 0.05). (E and F) HUVEC treated with control or NRP1 siRNA were treated with the indicated adenoviral vectors to compare their capacity to undergo tube formation in 3D collagen matrices after 72 hr. Representative images (E) and quantification (F) of tubulogenesis after 72 hr (mean ± SEM, n = 18, ^*^p < 0.05). Scale bar represents 100 um.

To define if impaired VEGF-A_165_ signaling in *Nrp1*^*cyto*^ EC is due to the absence of the PDZ-binding domain or another part of the NRP1 cytoplasmic tail, we transduced primary arterial EC from Nrp1^cyto^ mice with either a control adenoviral vector (*Ad*-*null*) or vectors expressing full-length NRP1 (*Ad*-*Nrp1*), NRP1 lacking the entire cytoplasmic domain (*Ad*-*Nrp1*^*cyto*^) or NRP1 lacking only the PDZ binding site (*Ad*-*Nrp1*^*PDZ*^). As NRP1^cyto^ expression levels are variable in vitro (but not in vivo), we chose cells with NRP1^cyto^ levels that best matched Nrp1 levels following transduction of various mutants noted above (Figure 4A). Although the full-length *Ad-Nrp1* construct restored VEGFR2 Y^1175^ phosphorylation and ERK activation to control levels, both *Ad*-*Nrp1*^*cyto*^ and *Ad*-*Nrp1*^*PDZ*^ were ineffective (Figures 4A and 4B). Similar results were obtained with EC from *Nrp1* conditional null (*Nrp1*^*fl/fl*^) mice that were transduced with a CRE-expressing vector (*Ad*-*Cre*) to ablate NRP1 expression and then transduced with *Ad-Nrp1*, *Ad-Nrp1*^*cyto*^, or *Ad-Nrp1*^*PDZ*^ (Figures 4C and 4D). Thus, only the full-length NRP1 construct restored VEGF-A_165_-dependent ERK activation in mutant cells to WT levels (Figures 4B and 4D). Together, these findings establish that the PDZ-binding region in the NRP1 cytoplasmic domain is essential for normal VEGFR2 and ERK activation by VEGF-A_165_ in arterial endothelial cells.

To link changes in ERK activation in *Nrp1*^*cyto*^ EC to the observed arteriogenesis defects, we used an in vitro 3D tubulogenesis assay with human umbilical cord endothelial cells (HUVEC) that mimics many features of arteriogenesis in vivo (Koh et al., 2008; Stratman and Davis, 2012; Stratman et al., 2011). Knocking down NRP1 with siRNA in this model significantly impaired tubulogenesis, whereas expression of NRP1, but not NRP1^PDZ^ or NRP1^cyto^ constructs, restored tubulogenesis (Figures 4E and 4F). Moreover, defective tubulogenesis in the presence of NRP1^cyto^ or NRP1^PDZ^ could be rescued by coexpression of constitutively active (CA) ERK (Figures 4E and 4F). Thus, restoring ERK activation is sufficient to restore tubulogenesis in *Nrp1*^*cyto*^ EC.

### Delayed VEGFR2 Trafficking in *Nrp1*^*cyto*^ Endothelial Cells

Previous studies demonstrated that reduced VEGFR2 endocytosis or delayed trafficking reduces its signaling output (Lanahan et al., 2010). Thus, loss of synectin delays VEGFR2 transit to early endosomes and reduces ERK signaling (Lanahan et al., 2010). Because NRP1 has been proposed to be involved in VEGFR2 trafficking (Salikhova et al., 2008; Simons, 2012; Simons and Eichmann, 2012; Ballmer-Hofer et al., 2011) and binds synectin through its cytoplasmic tail (Cai and Reed, 1999; Horowitz and Seerapu, 2012), we evaluated if the NRP1 cytoplasmic domain is essential for the cotrafficking of NRP1 and VEGFR2 in primary arterial EC.

**Figure 5:**
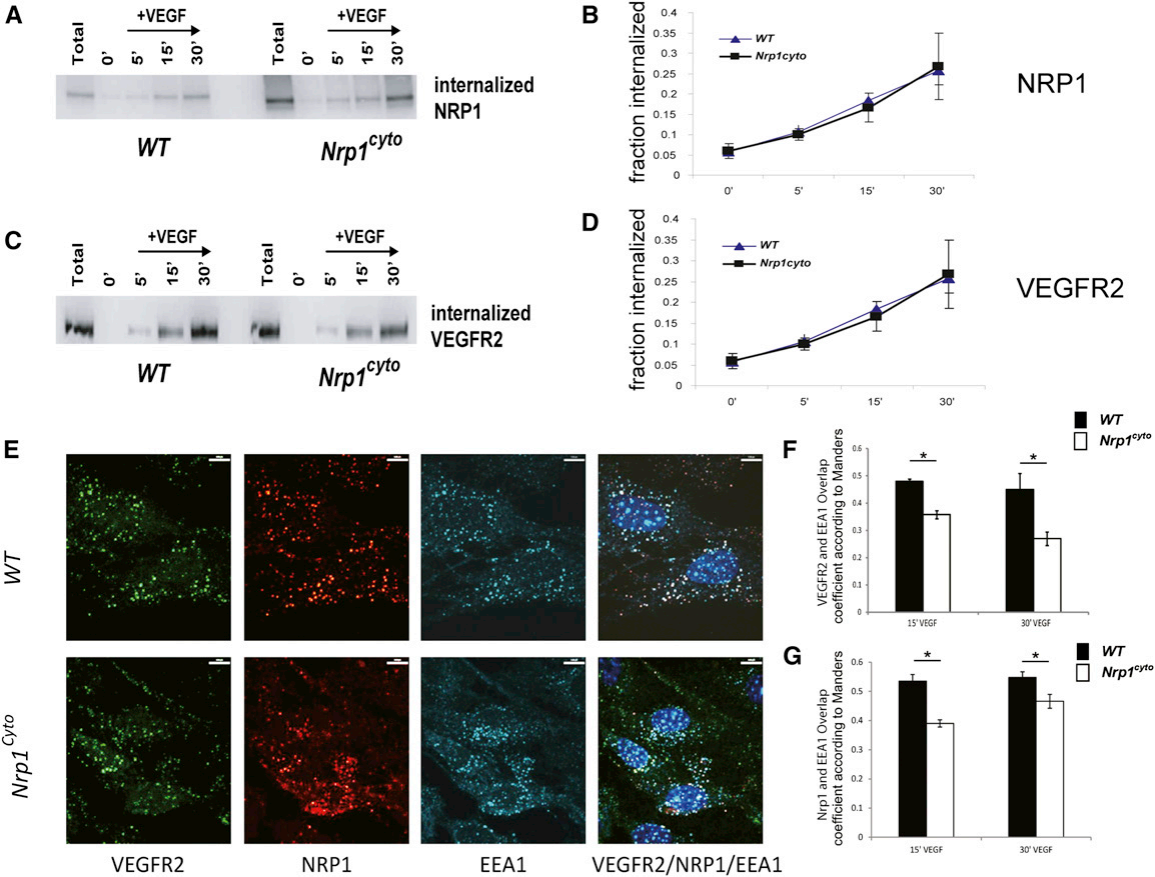
Normal Internalization but Reduced Colocalization of VEGFR2 and NRP1 in EAA1+ Endosomes in *Nrp1*^*cyto*^ Arterial EC (A–D) Western blot analysis of NRP1 and VEGFR2 internalization following cell surface biotinylation and VEGF-A_165_ stimulation in WT and *Nrp1*^*cyto*^ EC. (A and B) Blots show internalization at the indicated times. (C and D) Quantification of NRP1 and VEGFR2 internalization at 5, 15, and 30 min following VEGF-A_165_ stimulation relative to time 0 (mean ± SEM, n = 4). (E–G) VEGFR2 and NRP1 colocalization in EAA1+ endosomes in primary arterial EC from WT and *Nrp1*^*cyto*^ mice after VEGF-A_165_ stimulation. (E) Confocal images of EC that were immunolabeled for VEGFR2, NRP1, and EEA1 15 min after stimulation. Scale bar represents 9 μm. Quantification of VEGFR2 (F) and NRP1 (G) colocalization in EEA1-positive vesicles 15 and 30 min after VEGF-A_165_ stimulation shows a significant reduction in the number of EEA1 endosomes containing VEGFR2 in *Nrp1*^*cyto*^ relative to WT EC (overlap coefficient according to Manders, n = 10 independent fields for each time point and cell type, mean ± SEM, ^*^p < 0.05). See also Figure S5.

A time course analysis of biotinylated NRP1 in *Nrp1*^*cyto*^ and WT EC after exposure to VEGF-A_165_ showed a comparable extent and rate of NRP1 uptake (Figures 5A and 5B). The extent and rate of biotinylated VEGFR2 uptake was also similar in EC from both genotypes (Figures 5C and 5D). Confocal microscopy showed that prior to VEGF-A_165_ stimulation, VEGFR2 and NRP1 were distributed similarly on the plasma membrane (Figure S5A) and that the intracellular distribution of NRP1 (Figure S5B) in EC of both genotypes was also similar. In both control and *Nrp1*^*cyto*^ EC, VEGF-A_165_ stimulation led to accumulation of VEGFR2 and NRP1 in EEA1+ endosomes (Figure 5E). However, quantitative analysis demonstrated that the colocalization of VEGFR2 and NRP1 in EEA1+ endosomes was significantly reduced in *Nrp1*^*cyto*^ EC compared to WT EC at 15 min (Figures 5E–5G) and 30 min (Figures 5F, 5G, and S5C) after VEGF-A_165_ stimulation.

**Figure 6:**
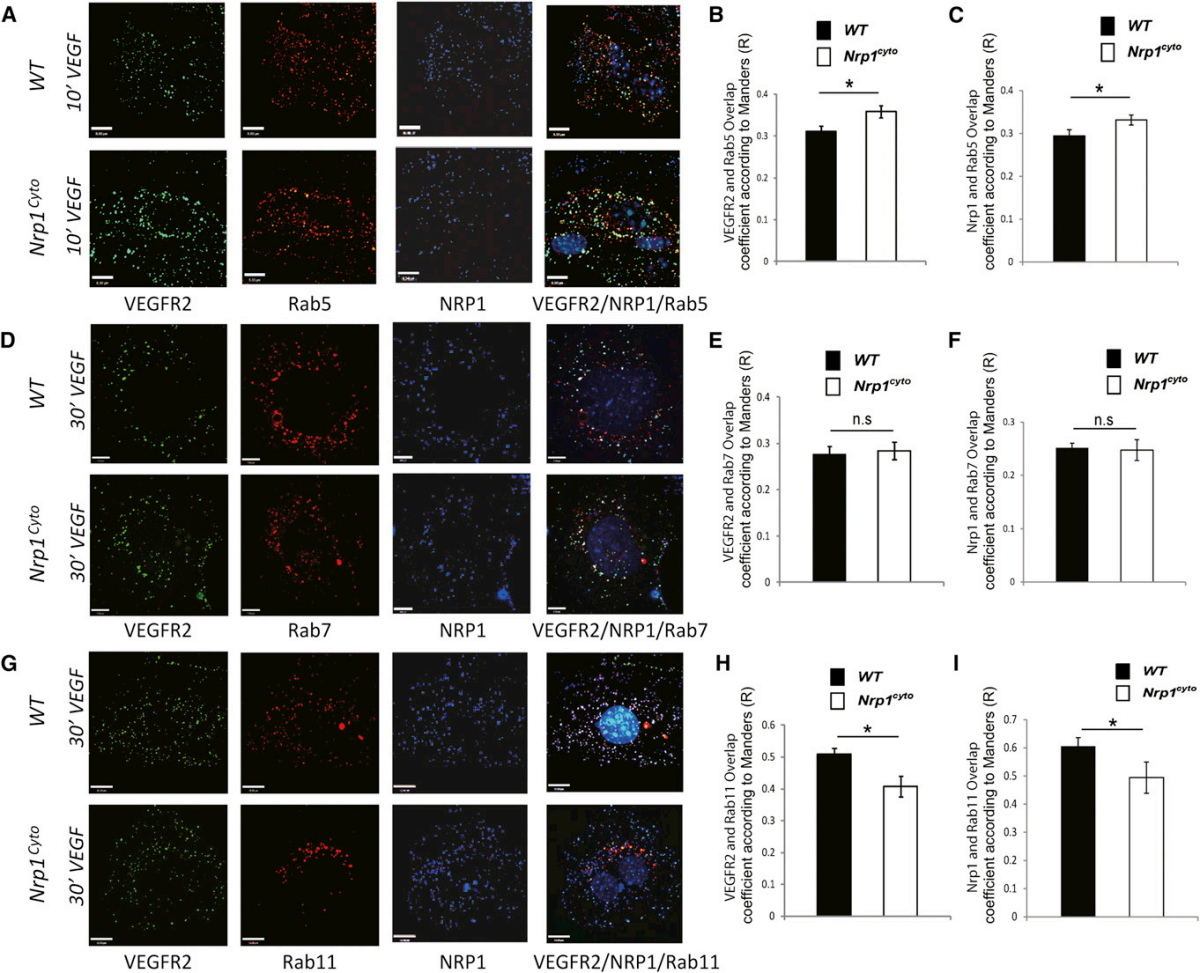
Impaired VEGFR2 and NRP1 Trafficking in *Nrp1*^*cyto*^ Arterial EC (A–C) Primary arterial EC from WT and *Nrp1*^*cyto*^ mice were immunolabeled for VEGFR2, NRP1, and Rab5 10 min after VEGF-A_165_ stimulation. Confocal images (A) (scale bar represents 9 μm) and quantification (B) and (C). The overlap coefficient according to Manders shows that the number of Rab5+ endosomes containing VEGFR2 and NRP1 is significantly increased in *Nrp1*^*cyto*^ relative to WT EC (n = 10 independent fields for each time point and cell type, mean ± SEM, ^*^p < 0.05). (D–F) Primary arterial EC from WT and *Nrp1*^*cyto*^ mice were immunolabeled for VEGFR2, NRP1, and Rab7 30 min after VEGF-A_165_ stimulation. Confocal images (D) (scale bar represents 7 μm) and quantification (E) and (F). The overlap coefficient according to Manders shows that the number of Rab7+ endosomes containing VEGFR2 and NRP1 is not changed in *Nrp1*^*cyto*^ relative to WT EC, even though both proteins accumulate in the upstream Rab5+ compartment in *Nrp1*^*cyto*^ EC (n = 10 independent fields for each time point and cell type, mean ± SEM). (G–I) Primary arterial EC from WT and *Nrp1*^*cyto*^ mice were immunolabeled for VEGFR2, NRP1, and Rab11 30 min after VEGF-A_165_ stimulation. Confocal images (G) (scale bar represents 12 μm) and quantification (H) and (I). The overlap coefficient according to Manders shows that the number of Rab11+ endosomes containing VEGFR2 and NRP1 is significantly decreased in *Nrp1*^*cyto*^ relative to WT EC, as expected, because both proteins accumulate in the upstream Rab5+ compartment in *Nrp1*^*cyto*^ EC (n = 10 independent fields for each time point and cell type, mean ± SEM, ^*^p < 0.05). See also Figures S6 and S7.

The reduced appearance of both VEGFR2 and NRP1 in EEA1+ endosomes of *Nrp1*^*cyto*^ EC after VEGF-A stimulation suggests that the complex either accumulates in a previous compartment (Rab5+ sorting endosomes) or moves faster to downstream compartments (Rab7+ and/or Rab11+ endosomes). To distinguish these possibilities, we compared colocalization of VEGFR2 and NRP1 in peripherally located Rab5+ endosomes 10 min after VEGF-A_165_ stimulation and in Rab7+ versus Rab11+ endosomes at 30 min after stimulation. At 10 min, the VEGFR2/NRP1 complex was present at significantly higher levels in Rab5+ endosomes in *Nrp1*^*cyto*^ compared to WT EC (Figures 6A–6C), suggesting slower clearance of the NRP1^cyto^/VEGFR2 compared to the NRP1/VEGFR2 complex from Rab5+ to EAA1+ endosomes. Even though VEGFR2 and NRP1 were delayed in upstream compartments, VEGFR2 and NRP1 were present at similar levels in Rab7+ endosomes at 30 min (Figures 6D–6F). These data are also consistent with previous observations, which suggested accelerated trafficking of VEGFR2 from EAA1 endosomes to Rab7+ endosomes in the absence of the NRP1 cytoplasmic tail (Ballmer-Hofer et al., 2011). Finally, VEGFR2 and NRP1 were present at higher levels in Rab11+ endosomes in WT compared to Nrp1^cyto^ EC (Figures 6G–6I), again consistent with a delay in trafficking from the Rab5 compartment.

To investigate whether the delayed trafficking of NRP1 and VEGFR2 from Rab5+ sorting to EAA1+ endosomes in *Nrp1*^*cyto*^ EC represents a general defect in clathrin-mediated endocytosis and trafficking, we studied the transferrin receptor as a prototypical membrane protein endocytosed via the clathrin pathway (Sorkin, 2004). We observed similar amounts of transferrin in EEA1+ endosomes in *Nrp1*^*cyto*^ and control EC (Figures S6A and S6B) demonstrating that the general process of clathrin-mediated endocytosis is normal in EC lacking the NRP1 cytoplasmic tail, and that reduced trafficking from Rab5+ to EAA1+ endosomes is specific to the VEGFR2/NRP1^cyto^ complex.

To demonstrate that full-length NRP1 is required for efficient VEGFR2 trafficking and that NRP1 accumulation in *Nrp1*^*cyto*^ EC was not responsible for the observed defects, we transduced mutant and control EC with *Ad*-*Nrp1*. We observed that 15 and 30 min after VEGF-A_165_ stimulation, the proportion of VEGFR2 in EEA1+ endosomes was significantly higher in *Ad-Nrp1* transduced *Nrp1*^*cyto*^ EC and similar to control EC (Figures S7A and S7B). These experiments demonstrate that full-length NRP1 rescues defective VEGFR2 trafficking in *Nrp1*^*cyto*^ EC.

### Delayed VEGFR2 Trafficking in *Nrp1*^*cyto*^ EC Reduces ERK Activation Due to PTP1b-Mediated Dephosphorylation of VEGFR2 Y1175

**Figure 7:**
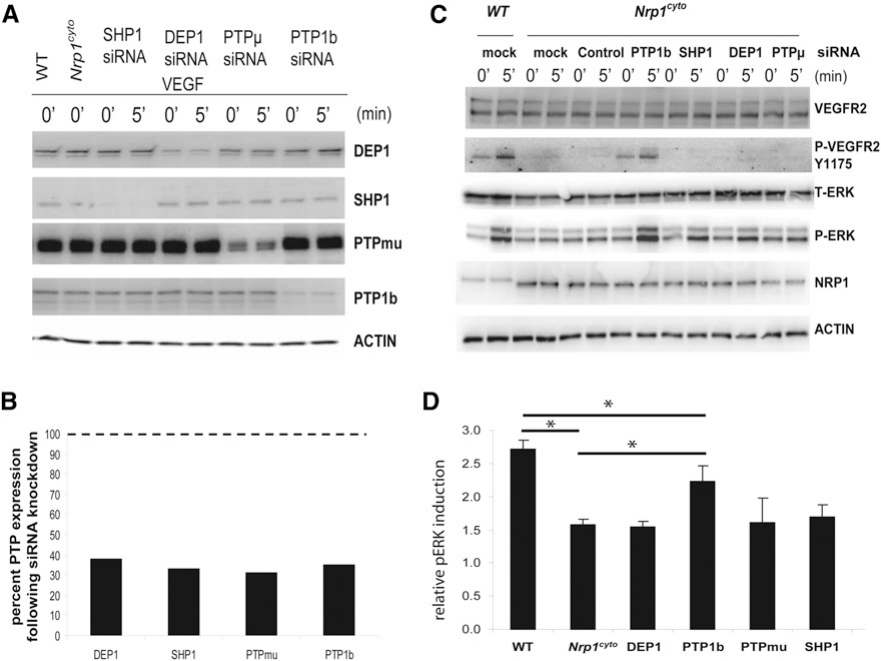
Rescue of Defective ERK Signaling in VEGF-A-Stimulated *Nrp1*^*cyto*^ Arterial EC by Knockdown of PTP1b Primary arterial EC from *Nrp1*^*cyto*^ mice transfected with siRNA specific for the indicated phosphatases were serum-starved and then stimulated with 50 ng/ml VEGF-A_165_. (A and B) Knockdown of the indicated phosphatases in *Nrp1*^*cyto*^ arterial EC shown by immunoblotting (A); PTP1b knockdown was quantified in (B), dashed line indicates normal expression levels. (C and D) ERK and VEGFR2 (Y1175) phosphorylation after knockdown of the indicated phosphatases shown by immunoblotting (C). Quantification of pERK activation is shown in (D) (n = 3, mean ± SD, ^*^p < 0.05).

We previously reported that delayed VEGFR2 trafficking in synectin null EC exposes the phosphorylated Y^1175^ site of VEGFR2 to dephosphorylation by PTP1b (Lanahan et al., 2010). Because *Nrp1*^*cyto*^ EC also demonstrated delayed VEGFR2 trafficking and reduced Y^1175^ phosphorylation, we knocked down PTP1b and several other phosphotyrosine phosphatases known to be involved in VEGF-A signaling in primary EC (Figures 7A and 7B). As observed for synectin null EC, knockdown of PTP1b in *Nrp1*^*cyto*^ EC restored Y^1175^ phosphorylation of VEGFR2 to normal levels (Figure 7C) and reactivated ERK signaling (Figures 7C and 7D). This effect was specific for PTP1b, as knockdown of several other protein phosphatases did not have this effect (Figures 7C and 7D).

These results suggest that PTP1b dephosphorylates VEGFR2 in *Nrp1*^*cyto*^ EC whereas it lingers in clathrin-coated vesicles or Rab5+ endosomes in *Nrp1*^*cyto*^ EC.

**Figure 8:**
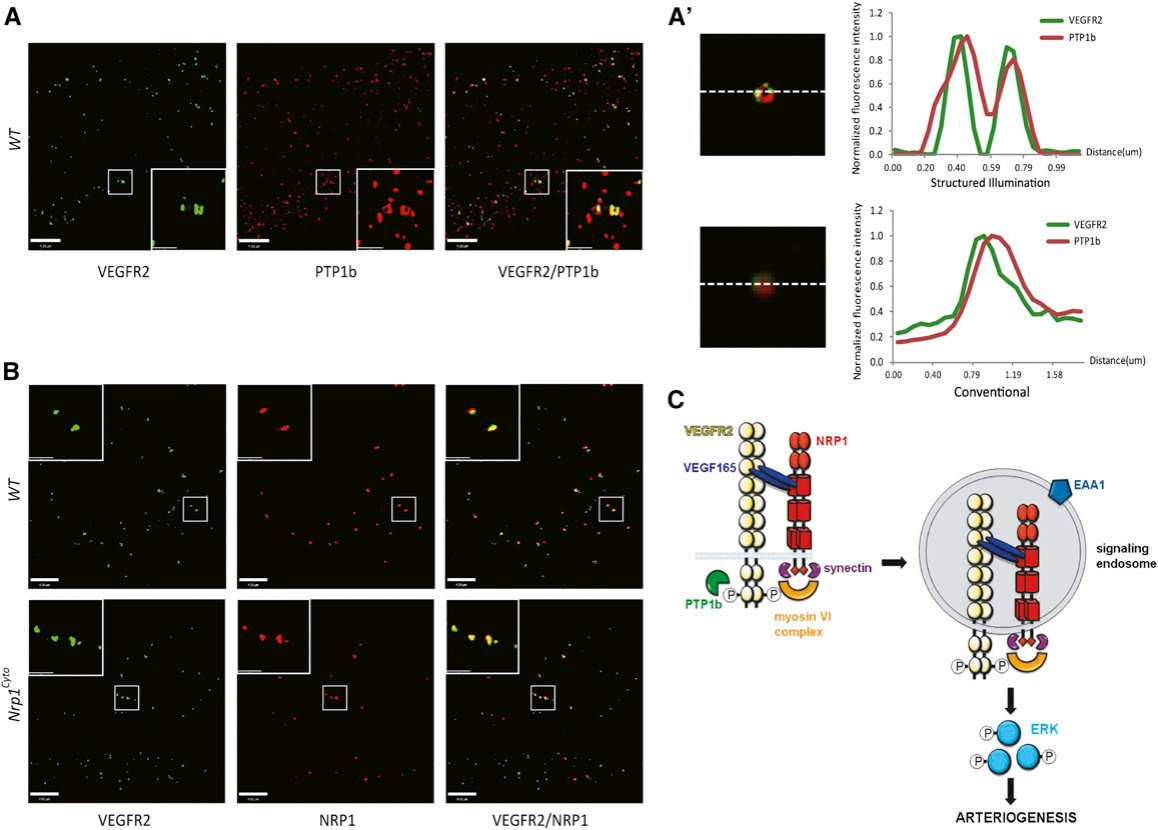
Colocalization of PTP1B, VEGFR2, and NRP1 by SIM Confocal Microscopy and Model of NRP1-Dependent Arteriogenic Signaling (A) SIM images of primary arterial EC immunolabeled for VEGFR2 and PTP1b 10 min after VEGF-A_165_ stimulation show colocalization of VEGFR2 and PTP1b in “donut”-shaped structures characteristic of early endosomes. (A′) The top panel shows an endocytic vesicle imaged using structured illumination microscopy and the adjacent graph plots localization of VEGFR2 and PTP1b in the vesicle. Note the colocalization of VEGFR2 and PTP1b in the donut-shaped structure characteristic of early endosomes. The bottom panel shows the same endocytic vesicle in (A), but imaged using conventional microscopy, and the adjacent graph plots localization of VEGFR2 and PTP1b in the vesicle; note the superior resolution of SIM. Scale bar represents 20 μm. (B) SIM images of primary arterial EC form WT and *Nrp1*^*cyto*^ mice immunolabeled for VEGFR2 and NRP1 15 min after VEGF-A_165_ stimulation show colocalization of VEGFR2 and NRP1 in both cell types. (C) Model of NRP1-dependent arteriogenic signaling. VEGF-A_165_ binding to VEGFR2 and NRP1 on the plasma membrane induces complex formation between the three molecules and leads to clathrin-mediated endocytosis. Phosphorylation of Y1175 in VEGFR2 stimulates ERK signaling, but is inactivated when exposed to the tyrosine phosphatase PTP1b at the plasma membrane or in RAB5+ endosomes. The NRP1 cytoplasmic domain links the VEGF-A_165_/VEGFR2/NRP1 complex to myosin VI via synectin to promote trafficking from RAB5+ to EAA1+ endosomes, where VEGFR2 maintains its Y1175 phosphorylation in the absence of exposure to PTP1b and is therefore able to evoke ERK signaling of sufficient amplitude to promote arteriogenesis. See also Figure S8.

Conventional confocal microscopy suggested colocalization of VEGFR2, NRP1, and PTP1b (Figure S8). We therefore used super resolution structured illumination microscopy (SIM) to examine whether VEGFR2, NRP1, and PTP1b colocalize to a common compartment. By employing a fine pattern of illumination stripes at well-defined positions and analyzing signal variation in fluorescence as a function of time and position, SIM achieves ∼8-fold improved volumetric resolution relative to conventional microscopy (Gustafsson et al., 2008; Toomre and Bewersdorf, 2010). In control EC, VEGFR2 and PTP1b localized to toroid-shaped structures close to the plasma cell membrane (Figures 8A and 8A′) that are characteristic of Rab5+ sorting endosome (Galperin et al., 2012; Galperin and Sorkin, 2003). Both NRP1 and NRP1^cyto^ colocalized with VEGFR2 in these structures (Figure 8B).

## Discussion

The results of this study demonstrate that the NRP1 cytoplasmic domain is dispensable for angiogenesis, but critical for normal arteriogenesis in both developmental and adult settings. The arteriogenic function of the NRP1 cytoplasmic domain was traced to an essential role in VEGFR2 trafficking and further shown to require PDZ-dependent interactions. The observed trafficking defect in EC lacking the NRP1 cytoplasmic domain was characterized by delayed trafficking of VEGFR2 from Rab5+ sorting to EEA1+ endosomes, leading to increased PTP1b-dependent dephosphorylation of the VEGFR2 Y^1175^ site that is required for activation of the PLCγ/ERK pathway. The reintroduction of NRP1 with an intact cytoplasmic domain or the suppression of PTP1b expression fully restored VEGFR2 trafficking and VEGF-A-dependent ERK activation.

In a previous study, we demonstrated that the absence of the NRP1 PDZ-binding partner synectin led to similar developmental and adult arterial morphogenesis defects, decreased VEGFR2 trafficking, and reduced ERK activation; these defects were also correctable by suppression of PTP1b activity or expression (Lanahan et al., 2010). Taken together, these findings establish that NRP1 and synectin together promote the proper movement of VEGFR2 to EEA1+ endosomes and that this is a key step in regulating VEGF-A-dependent ERK activation.

VEGF-A signaling has long been recognized as critical for all aspects of vascular development including vasculogenesis, angiogenesis, and arteriogenesis. Thus, deletion of VEGF-A, VEGFR2, or PLCγ, an enzyme critical for VEGFR2-dependent ERK activation, results in early embryonic lethality (Chung and Ferrara, 2011). Given the central role played by VEGF-A signaling in the vasculature, it is not surprising that it is tightly controlled at multiple levels. Recent studies have firmly established that receptor tyrosine kinase signaling is not extinguished after receptor-ligand complex internalization from the plasma membrane, but continues in various endosomal compartments (Dobrowolski and De Robertis, 2012; Platta and Stenmark, 2011). VEGFR2 uptake proceeds in a classical clathrin- and dynamin-dependent fashion, with much of ERK signaling occurring in EEA1+ endosomes (Germain and Eichmann, 2010). Upon endocytosis, VEGFR2 is initially found in clathrin-coated vesicles, moves on to Rab5+ sorting endosomes, and then transitions to EEA1+ endosomes, an intracellular trafficking process that utilizes synectin/myosin VI complexes (Eichmann and Simons, 2012). The previous puzzle surrounding VEGFR2 trafficking was how a VEGFR2-containing endosome could be coupled to the synectin/myosin-VI motor, given the absence of a PDZ binding domain in the receptor. Here we show that this role is played by NRP1 via its PDZ-based interaction with synectin.

Synectin has two principle protein interaction sites, a PDZ2 and a myosin VI binding domain. Through the PDZ2 site, synectin interacts not only with NRP1, but also with other transmembrane proteins, such as syndecan-4 and the TRK receptors (De Vries et al., 1998; Gao et al., 2000; Lou et al., 2001). These interactions promote specific signaling events, such as syndecan 4-dependent activation of RhoG and Rac1 (Elfenbein et al., 2009) and IGF1-dependent ERK activation (Booth et al., 2002; Lou et al., 2001) in EC or BDNF-stimulated synaptic transmission in hippocampal neurons (Yano et al., 2006). By binding myosin VI, synectin then mediates inward endosomal vesicle trafficking in a number of different cell types, including EC (Naccache et al., 2006; Varsano et al., 2006). Because synectin/myosin-VI-mediated trafficking is particularly important to promote VEGFR2 signaling in arterial EC, the knockout of either synectin or myosin VI results in arterial-specific morphogenic defects characterized by a smaller number of arteries, decreased lumen sizes, and reduced branching complexity of the arterial tree, both during development and in adult tissues (Chittenden et al., 2006; Lanahan et al., 2010).

Mechanistically, impaired VEGFR2 trafficking in synectin null EC leads to prolonged retention of VEGFR2 in a previously unidentified cytoplasmic compartment containing PTP1b, a phosphatase that targets the Y^1175^ site of VEGFR2 to reduce ERK activation. As VEGFR2 cannot directly interact with synectin to facilitate its trafficking away from PTP1b, another molecule must be involved. A likely candidate has been NRP1, as it interacts with both VEGFR2 and synectin biochemically (Cai and Reed, 1999; Soker et al., 2002), and because NRP1-VEGFR2 complex formation on the plasma cell membrane of EC in vitro requires the PDZ-binding domain of NRP1 (Prahst et al., 2008). Our study provides direct evidence that the interaction of NRP1 with both VEGFR2 and synectin is functionally significant and specifically important for arteriogenesis.

A previous study in EC that overexpressed VEGFR2 and NRP1 supports a critical role for the NRP1 cytoplasmic domain in mediating VEGFR2 trafficking (Ballmer-Hofer et al., 2011). In these experiments, the NRP1-binding isoform of VEGF-A, VEGF-A_165a_, directed VEGFR2 to the Rab5/Rab4/Rab11 recycling pathway in a NRP1-dependent fashion, whereas the non-NRP1-binding isoform VEGF-A_165b_ directed VEGFR2 toward degradation via the Rab7 pathway. Moreover, NRP1 targeting of endocytosed VEGFR2 to the Rab5/Rab11 pathway requires the presence of the NRP1 PDZ binding domain (Salikhova et al., 2008), suggesting a synectin-dependent process. We have now extended these observations by demonstrating that NRP1 promotes the transition of VEGFR2 from Rab5+ sorting to EAA1+ endosomes in a pathway that requires the NRP1 synectin-binding domain to stimulate ERK signaling for arteriogenesis. Thus, the reduced appearance of VEGFR2/NRP1 complexes in EEA1+ endosomes is due to a combination of an upstream defect, a delay in trafficking from Rab5+ sorting endosomes, and a downstream defect, the increased clearance of the complex to Rab7+, but not Rab11+ compartments.

We further confirmed with SIM imaging that the endocytosed VEGFR2/NRP1 complex is found in a complex with PTP1b in Rab5+ sorting endosomes that have a characteristic toroid appearance, i.e., before it enters EAA1+ endosomes. The reduced level of VEGFR2 phosphorylation on Y^1175^ we observe in *Nrp1*^*cyto*^ EC can therefore be explained by prolonged exposure to PTP1b and agrees with the kinetics of delayed VEGFR2 trafficking in synectin null EC (Lanahan et al., 2010) and the fact that PTP1b knockdown restored VEGFR2 Y1175 phosphorylation and ERK activation in both types of mutants.

Reduced VEGF-A-dependent ERK activation in primary EC and mice lacking the NRP1 cytoplasmic tail was associated with both developmental and adult arteriogenic defects in vivo and impaired tubulogenesis in vitro. The in vitro tubulogenesis assay offered additional mechanistic insight into NRP1-dependent VEGFR2 trafficking and ERK activation. Thus, reduced ERK activation in EC expressing NRP1^cyto^ could be corrected by expressing full-length NRP1, but not NRP1 lacking the PDZ domain, consistent with the idea that this domain regulates VEGFR2 trafficking and ERK signaling via synectin/myosin-VI recruitment. Moreover, constitutively active ERK effectively restored tubulogenesis in EC expressing the NRP1^cyto^ mutant protein. Taken together, these observations support the hypothesis that NRP1/VEGFR2 trafficking-dependent ERK activation is critical for vascular tubulogenesis in vitro and, by extension, arteriogenesis in vivo.

Overall, this study supports the concept that robust NRP1/VEGFR2-mediated ERK activation from the EAA1+ endosomes is central to arterial morphogenesis, but not angiogenesis. Other data supporting this notion include the direct dependence of arterial morphogenesis in *gridlock* zebrafish on the extent of ERK signaling (Hong et al., 2008; Hong et al., 2006), reduced arteriogenesis in fish and mice after synectin and myosin-VI knockdown (Chittenden et al., 2006; Dedkov et al., 2007; Lanahan et al., 2010), and the ability of a constitutively active ERK mutant to overcome VEGF-A signaling defects in synectin null mice and therefore restore normal arterial morphogenesis (Ren et al., 2010).

In view of these finding, we suggest that VEGF-A regulates angiogenesis and arteriogenesis via two distinctly different pathways. Angiogenesis is controlled by VEGF-A-induced VEGFR2 signaling events that do not require synectin/myosin-VI-dependent endocytosis and trafficking of the VEGFR2/NRP1 complex. This happens either because it requires lower levels of ERK activation than arteriogenesis or because it is more reliant on other VEGF-initiated signaling events at the plasma membrane such as, for example, PI3K/AKT activation. Arteriogenesis, however, is critically dependent on high level endothelial ERK activation and therefore requires VEGF-A_165_-induced NRP1 and VEGFR2 coendocytosis and subsequent trafficking of the VEGFR2/NRP1/synectin/myosin-VI complex away from the plasma membrane and PTP1b associated with Rab5 sorting endosomes to ensure prolonged ERK signaling from EEA1+ endosomes (Figure 8C).

## Experimental Procedures

### Animals

Mice lacking the cytoplasmic tail of NRP1 (*Nrp1*^*cyto*^) have previously been described (Fantin et al., 2011). All animal husbandry and experiments were performed in compliance with the guidelines established by institutional review boards.

### Reagents and Antibodies

The following regents were used: transferrin from human serum, Alexa Fluor 488 conjugate (Invitrogen T-13342), human fibronectin (BD Biosciences 356008), VEGF-A_165_ (293-VE R&D Systems), PTP siRNAs FlexiTube (R-PTP-mu [PTPRM], DEP1, PTP1B, SHP1 (PTPN6); QIAGEN 1027416), EZ-Link Sulfo-NHS-SS-Biotin (Pierce 21331), and immobilized Neutravidin protein (Pierce 29200). Antibodies include rat monoclonal VEGFR2 (Flk-1, clone Avas12α1 Fitzgerald 10R-6549), goat polyclonal NRP1 (R&D Systems AF566), goat polyclonal NRP1 (Santa Cruz 7239), goat polyclonal anti-EEA1 (Santa Cruz 6415), rabbit polyclonal anti-EEA1 (Santa Cruz 33585), rabbit monoclonal Rab5 (Cell Signaling 3547), rabbit monoclonal Rab7 (Cell Signaling 9367), goat polyclonal anti-VE-cadherin (Santa Cruz SC6458), anti-phospho-VEGFR2 (Tyr1175, Cell Signaling 2478), anti-total VEGFR2 (Cell Signaling 2479), anti-phospho p44/42 MAP kinase (phospho-ERK, Cell Signaling 9106), anti-p44/42 MAP kinase (total ERK, Cell Signaling 9102), anti-PTP1b (Upstate 07-088, BD 610139), anti-DEP-1/CD148 (R&D AF1934), anti-PTPmu (Cell Signaling 4485), and anti-SHP1 (Cell Signaling 3759). We used appropriate secondary antibodies that were conjugated to horseradish peroxidase (Vector Laboratories) or fluorescently labeled (Life Technologies).

### Adenovirus Rescue of Floxed *Nrp1* and *Nrp1*^*cyto*^ Endothelial Cells

Floxed *Nrp1* EC were grown to 60%–70% confluence on gelatin-coated dishes and treated with Cre adenovirus overnight in media containing 20% FBS at a multiplicity of infection of 100. The washed cells or *Nrp1*^*cyto*^ EC were then treated with control CMV, *Nrp1*^*cyto*^, *Nrp1*^*PDZ*^, or full-length NRP1 overnight, washed, grown an additional 2 days, and then starved overnight in 0.5% FBS. Cells were stimulated for 5 min with 50 ng/ml VEGF-A_165_ and lysed in NP40 buffer.

### siRNA Transfection of Endothelial Cells

EC were grown to 60–70% confluence on gelatin-coated dishes and treated on 2 consecutive days for 4 hr with 200 pmol/dish of siRNA (QIAGEN FlexiTube siRNA or Santa Cruz siRNA) in 2 ml growth media containing 0.5% FBS and TransPass R2 transfection reagent-siRNA suspension (New England BioLabs). EC were then starved overnight in growth media containing 0.5% FBS, stimulated for 5 min with 50 ng/mlVEGF-A_165_ and lysed in NP40 buffer.

### Colocalization of Transferrin and EEA1

Cells were starved overnight with 0.5% FBS, placed on ice for 20 min, incubated with Alexa Fluor488 conjugated transferrin on ice for 30 min, rinsed three times with cold PBS and then stimulated with serum at 37°C for various times. After rinsing twice with ice cold PBS pH 2.5, cells were fixed at room temperature in 4% formaldehyde in PBS for 10 min and permeabilized with 2.5% formaldehyde containing 0.1% Triton X-100 and 0.1% NP-40 for 10 min at room temperature. Cells were then labeled with rabbit anti-EEA1 followed by chicken anti-rabbit Alexa 568 antibody. Samples were mounted with ProLong Gold DAPI (Life Technologies) and imaged using an UltraVIEW VoX spinning disc confocal microscope with a 60× oil immersion lens (Perkin Elmer) and equipped with a Ti-E microscope stand (Nikon). Image analysis was performed using the colocalization module of Volocity software.

### Colocalization of VEGFR2, NRP1, and EEA1 or Rab5, Rab7, Rab11, and PTP1b

Cells were plated on gelatin-coated glass bottom dishes (MatTek), starved overnight with 0.5% FBS, and then placed on ice. Cells were incubated with 10 μg/ml rat anti-mouse VEGFR2 and goat anti-rat NRP1 for 15 min and rinsed three times with cold PBS. Cells were stimulated with 50 ng/mlVEGF-A_165_, incubated at 37°C for various times, washed twice with ice cold PBS pH 2.5, rinsed three times with cold PBS and then fixed for 10 min at room temperature in 4% formaldehyde in PBS and permeabilized with 2.5% formaldehyde containing 0.1% Triton X-100 and 0.1% NP-40 for 10 min at room temperature. Cells were labeled with rabbit anti-EEA1 followed by chicken anti-rabbit Alexa647 secondary antibody. For detection of VEGFR2 and NRP1, cells were labeled with chick anti-rat alexa488 and donkey anti-goat alexa568 secondary antibodies. For Rab5, Rab7, and PTP1b costaining, NRP1 primary antibody was labeled with chick anti-goat alexa647, whereas primary antibodies for Rab5 or Rab7 were labeled with anti-rabbit alexa568, and PTP1b with anti-mouse alexa568. For Rab11 staining, a Rab11-RFP adenovirus was used to transduce cells prior to labeling. Samples were mounted with ProLong Gold DAPI and imaged with a Perkin Elmer UltraVIEW VoX spinning disc confocal microscope equipped with a 60× oil immersion lens and a 14 bit electron-multiplied, charge coupled device camera (8 μm pixel size; Hamamatsu C9100-50). Thresholds for z stack images were acquired using the MaxEntropy automatic threshold method provided by the ImageJ MultiThresholder plugin of (NIH Bethesda) (Baler, Landini & Rasband). The overlap coefficient according to Manders was calculated using the ImageJ colocalization analysis plugin JACoP (Bolte & Cordelieres).

### SIM Microscopy

Images were acquired using a U-PLANAPO 60×/1.42 PSF, oil immersion objective lens (Olympus, Center Valley, PA) and CoolSNAP HQ^2^ CCD cameras with a pixel size of 0.080 μm (Photometrics, Tucson, AZ) on the OMX version 3 system (Applied Precision) equipped with 488, 561, and 642 nm solid-state lasers (Coherent and MPB communications). Samples were illuminated by a coherent scrambled laser light source that had passed through a diffraction grating to generate the structured illumination by interference of light orders in the image plane to create a 3D sinusoidal pattern, with lateral stripes approximately 0.270 nm apart. The pattern was shifted laterally through five phases and through three angular rotations of 60° for each z section, separated by 0.125 nm. Exposure times were typically between 200 and 500 ms, and the power of each laser was adjusted to achieve optimal intensities of between 2,000 and 4,000 counts in a raw image of 16-bit dynamic range, at the lowest possible laser power to minimize photo bleaching. Raw images were processed and reconstructed to reveal structures with 100–125 nm resolution (Gustafsson et al., 2008). The channels were then aligned in x, y, and rotationally using predetermined shifts as measured using a target lens and the Softworx alignment tool (Applied Precision).

### In Vitro Three-Dimensional Endothelial Lumen Formation Assay

As previously described, human umbilical cord EC were cultured in 3D collagen matrices (2.5 mg/ml) at 2 × 10^6^/ml to assess their ability to form lumens and tubes (Koh et al., 2008). EC were treated twice with 20 nM NRP1 or control siRNA over a 3-day period and then overnight with adenoviral vectors expressing LacZ control, CA ERK, NRP1, NRP1^cyto^, or NRP1^PDZ^. Collagen gels contained 200 ng/ml recombinant stem cell factor, interleukin-3, stromal-derived factor 1α, and FGF-2 (Stratman et al., 2011). The culture media contained VEGF-A_165_ and FGF-2 at 40 ng/ml and reduced serum-supplement II as described (Koh et al., 2008). After 3 days, cultures were fixed with glutaraldehyde, stained with toluidine blue, and photographed. EC lumen and tube areas were quantitated from these photographs by tracing total lumen area in each field using Metamorph software as described (Koh et al., 2008).

### Whole-Mount Immunolabeling

As previously described (Fantin et al., 2013), dissected E12.5 hindbrain tissue was fixed in with 4% formaldehyde in PBS on ice, washed, incubated with serum-free protein block (Dako), and immunolabeled with goat anti-rat NRP1, goat anti-VEGFR2 (R&D Systems AF566, AF644) or rabbit anti-neurofilament (Millipore AB1981) followed by Alexa488-conjugated goat anti-rabbit IgG (Molecular Probes) and Cy3-conjugated rabbit anti-goat Fab fragment (Jackson Immuno). Samples were imaged with a LSM710 laser scanning confocal microscopes (Zeiss).

### Alkaline Phosphatase Fusion Protein Binding Assay

As previously described (Fantin et al., 2013), alkaline phosphatase (AP)-VEGF_165_ fusion protein was incubated with freshly dissected E12.5 hindbrain tissue, washed three times for 20 min each with PBS, fixed with 4% formaldehyde in PBS for 1 hr, and washed again. Endogenous AP was heat-inactivated at 65°C for 3 hr. Tissue-bound, heat-stable, recombinant AP activity was detected as an insoluble reaction product after incubation with nitro blue tetrazolium and 5-bromo-4-chloro-3-indolyl phosphate (Roche) in a buffer containing 100 mM Tris pH 9.5, 100 mM NaCl, and 5 mM MgCl_2_. Images were recorded using an MZ16 microscope (Leica) equipped with a Micropublisher 3.3 camera (Q Imaging).

### Statistical Analysis

All data are shown as mean ± SEM or SD as bars in the histograms. Differences were considered statistically significant if p ≤ 0.05 by Student’s t test.

## Supplemental Information

Document S1. Figures S1–S8 and Supplemental Experimental Procedures

## References

[bib1] Ballmer-Hofer K., Andersson A.E., Ratcliffe L.E., Berger P. (2011). Neuropilin-1 promotes VEGFR-2 trafficking through Rab11 vesicles thereby specifying signal output. Blood.

[bib2] Booth R.A., Cummings C., Tiberi M., Liu X.J. (2002). GIPC participates in G protein signaling downstream of insulin-like growth factor 1 receptor. J. Biol. Chem..

[bib3] Cai H., Reed R.R. (1999). Cloning and characterization of neuropilin-1-interacting protein: a PSD-95/Dlg/ZO-1 domain-containing protein that interacts with the cytoplasmic domain of neuropilin-1. J. Neurosci..

[bib4] Carmeliet P., Jain R.K. (2011). Molecular mechanisms and clinical applications of angiogenesis. Nature.

[bib5] Chittenden T.W., Claes F., Lanahan A.A., Autiero M., Palac R.T., Tkachenko E.V., Elfenbein A., Ruiz de Almodovar C., Dedkov E., Tomanek R. (2006). Selective regulation of arterial branching morphogenesis by synectin. Dev. Cell.

[bib6] Chung A.S., Ferrara N. (2011). Developmental and pathological angiogenesis. Annu. Rev. Cell Dev. Biol..

[bib7] De Vries L., Lou X., Zhao G., Zheng B., Farquhar M.G. (1998). GIPC, a PDZ domain containing protein, interacts specifically with the C terminus of RGS-GAIP. Proc. Natl. Acad. Sci. USA.

[bib8] Dedkov E.I., Thomas M.T., Sonka M., Yang F., Chittenden T.W., Rhodes J.M., Simons M., Ritman E.L., Tomanek R.J. (2007). Synectin/syndecan-4 regulate coronary arteriolar growth during development. Dev. Dyn..

[bib9] Dobrowolski R., De Robertis E.M. (2012). Endocytic control of growth factor signalling: multivesicular bodies as signalling organelles. Nat. Rev. Mol. Cell Biol..

[bib10] Eichmann A., Simons M. (2012). VEGF signaling inside vascular endothelial cells and beyond. Curr. Opin. Cell Biol..

[bib11] Elfenbein A., Rhodes J.M., Meller J., Schwartz M.A., Matsuda M., Simons M. (2009). Suppression of RhoG activity is mediated by a syndecan 4-synectin-RhoGDI1 complex and is reversed by PKCalpha in a Rac1 activation pathway. J. Cell Biol..

[bib12] Fantin A., Schwarz Q., Davidson K., Normando E.M., Denti L., Ruhrberg C. (2011). The cytoplasmic domain of neuropilin 1 is dispensable for angiogenesis, but promotes the spatial separation of retinal arteries and veins. Development.

[bib13] Fantin A., Vieira J.M., Plein A., Maden C.H., Ruhrberg C. (2013). The embryonic mouse hindbrain as a qualitative and quantitative model for studying the molecular and cellular mechanisms of angiogenesis. Nat. Protoc..

[bib14] Galperin E., Sorkin A. (2003). Visualization of Rab5 activity in living cells by FRET microscopy and influence of plasma-membrane-targeted Rab5 on clathrin-dependent endocytosis. J. Cell Sci..

[bib15] Galperin E., Abdelmoti L., Sorkin A. (2012). Shoc2 is targeted to late endosomes and required for Erk1/2 activation in EGF-stimulated cells. PLoS ONE.

[bib16] Gao Y., Li M., Chen W., Simons M. (2000). Synectin, syndecan-4 cytoplasmic domain binding PDZ protein, inhibits cell migration. J. Cell. Physiol..

[bib17] Germain S., Eichmann A. (2010). VEGF and ephrin-B2: a bloody duo. Nat. Med..

[bib18] Gustafsson M.G., Shao L., Carlton P.M., Wang C.J., Golubovskaya I.N., Cande W.Z., Agard D.A., Sedat J.W. (2008). Three-dimensional resolution doubling in wide-field fluorescence microscopy by structured illumination. Biophys. J..

[bib19] Hong C.C., Peterson Q.P., Hong J.Y., Peterson R.T. (2006). Artery/vein specification is governed by opposing phosphatidylinositol-3 kinase and MAP kinase/ERK signaling. Curr. Biol..

[bib20] Hong C.C., Kume T., Peterson R.T. (2008). Role of crosstalk between phosphatidylinositol 3-kinase and extracellular signal-regulated kinase/mitogen-activated protein kinase pathways in artery-vein specification. Circ. Res..

[bib21] Horowitz A., Seerapu H.R. (2012). Regulation of VEGF signaling by membrane traffic. Cell. Signal..

[bib22] Koch S. (2012). Neuropilin signalling in angiogenesis. Biochem. Soc. Trans..

[bib23] Koch S., Tugues S., Li X., Gualandi L., Claesson-Welsh L. (2011). Signal transduction by vascular endothelial growth factor receptors. Biochem. J..

[bib24] Koh W., Stratman A.N., Sacharidou A., Davis G.E. (2008). In vitro three dimensional collagen matrix models of endothelial lumen formation during vasculogenesis and angiogenesis. Methods Enzymol..

[bib25] Lanahan A.A., Hermans K., Claes F., Kerley-Hamilton J.S., Zhuang Z.W., Giordano F.J., Carmeliet P., Simons M. (2010). VEGF receptor 2 endocytic trafficking regulates arterial morphogenesis. Dev. Cell.

[bib26] Lin D.C., Quevedo C., Brewer N.E., Bell A., Testa J.R., Grimes M.L., Miller F.D., Kaplan D.R. (2006). APPL1 associates with TrkA and GIPC1 and is required for nerve growth factor-mediated signal transduction. Mol. Cell. Biol..

[bib27] Lou X., Yano H., Lee F., Chao M.V., Farquhar M.G. (2001). GIPC and GAIP form a complex with TrkA: a putative link between G protein and receptor tyrosine kinase pathways. Mol. Biol. Cell.

[bib28] Naccache S.N., Hasson T., Horowitz A. (2006). Binding of internalized receptors to the PDZ domain of GIPC/synectin recruits myosin VI to endocytic vesicles. Proc. Natl. Acad. Sci. USA.

[bib29] Platta H.W., Stenmark H. (2011). Endocytosis and signaling. Curr. Opin. Cell Biol..

[bib30] Prahst C., Héroult M., Lanahan A.A., Uziel N., Kessler O., Shraga-Heled N., Simons M., Neufeld G., Augustin H.G. (2008). Neuropilin-1-VEGFR-2 complexing requires the PDZ-binding domain of neuropilin-1. J. Biol. Chem..

[bib31] Ren B., Deng Y., Mukhopadhyay A., Lanahan A.A., Zhuang Z.W., Moodie K.L., Mulligan-Kehoe M.J., Byzova T.V., Peterson R.T., Simons M. (2010). ERK1/2-Akt1 crosstalk regulates arteriogenesis in mice and zebrafish. J. Clin. Invest..

[bib32] Salikhova A., Wang L., Lanahan A.A., Liu M., Simons M., Leenders W.P., Mukhopadhyay D., Horowitz A. (2008). Vascular endothelial growth factor and semaphorin induce neuropilin-1 endocytosis via separate pathways. Circ. Res..

[bib33] Simons M. (2008). Chapter 14. Assessment of arteriogenesis. Methods Enzymol..

[bib34] Simons M. (2012). An inside view: VEGF receptor trafficking and signaling. Physiology (Bethesda).

[bib35] Simons M., Eichmann A. (2012). “On-target” cardiac effects of anticancer drugs: lessons from new biology. J. Am. Coll. Cardiol..

[bib36] Smith L.E., Wesolowski E., McLellan A., Kostyk S.K., D’Amato R., Sullivan R., D’Amore P.A. (1994). Oxygen-induced retinopathy in the mouse. Invest. Ophthalmol. Vis. Sci..

[bib37] Soker S., Miao H.Q., Nomi M., Takashima S., Klagsbrun M. (2002). VEGF165 mediates formation of complexes containing VEGFR-2 and neuropilin-1 that enhance VEGF165-receptor binding. J. Cell. Biochem..

[bib38] Sorkin A. (2004). Cargo recognition during clathrin-mediated endocytosis: a team effort. Curr. Opin. Cell Biol..

[bib39] Sorkin A., von Zastrow M. (2009). Endocytosis and signalling: intertwining molecular networks. Nat. Rev. Mol. Cell Biol..

[bib40] Stratman A.N., Davis G.E. (2012). Endothelial cell-pericyte interactions stimulate basement membrane matrix assembly: influence on vascular tube remodeling, maturation, and stabilization. Microsc. Microanal..

[bib41] Stratman A.N., Davis M.J., Davis G.E. (2011). VEGF and FGF prime vascular tube morphogenesis and sprouting directed by hematopoietic stem cell cytokines. Blood.

[bib42] Tonnesen M.G., Feng X., Clark R.A. (2000). Angiogenesis in wound healing. J. Investig. Dermatol. Symp. Proc..

[bib43] Toomre D., Bewersdorf J. (2010). A new wave of cellular imaging. Annu. Rev. Cell Dev. Biol..

[bib44] Varsano T., Dong M.Q., Niesman I., Gacula H., Lou X., Ma T., Testa J.R., Yates J.R., Farquhar M.G. (2006). GIPC is recruited by APPL to peripheral TrkA endosomes and regulates TrkA trafficking and signaling. Mol. Cell. Biol..

[bib45] Vieira J.M., Schwarz Q., Ruhrberg C. (2007). Selective requirements for NRP1 ligands during neurovascular patterning. Development.

[bib46] Wang L., Mukhopadhyay D., Xu X. (2006). C terminus of RGS-GAIP-interacting protein conveys neuropilin-1-mediated signaling during angiogenesis. FASEB J..

[bib47] Yano H., Ninan I., Zhang H., Milner T.A., Arancio O., Chao M.V. (2006). BDNF-mediated neurotransmission relies upon a myosin VI motor complex. Nat. Neurosci..

