# Supplementary material for: The Neuropilin 1 Cytoplasmic Domain Is Required for VEGF-A-Dependent Arteriogenesis
Source: Dev Cell. 2013 Apr 29;25(2):156–68. doi: 10.1016/j.devcel.2013.03.019 (PMC3774154; doi:10.1016/j.devcel.2013.03.019)
Supplement: Document S1. Figures S1–S8 and Supplemental Experimental Procedures [file mmc1.pdf]

Developmental Cell, Volume *25*

Supplemental Information

The Neuropilin 1 Cytoplasmic Domain Is Required for VEGF-A-Dependent Arteriogenesis

Anthony Lanahan, Xi Zhang, Alessandro Fantin, Zhen Zhuang, Felix Rivera-Molina, Katherine Speichinger, Claudia Prahst, Jiasheng Zhang, Yingdi Wang, George Davis, Derek Toomre, Christiana Ruhrberg, and Michael Simons

Inventory of Supplemental Information

Supplemental Figures and Figure Legends
Figure S1, related to Figure 1. Loss of the NRP1 cytoplasmic domain does not impair pathological angiogenesis.
Figure S2, related to Figure 1. Body weights of wildtype and $Nrp1^{cyto}$ mice, and heart and kidney tissues.
Figure S3, related to Figure 2. Synectin and pERK expression in $Nrp1^{cyto}$ and WT mice following femoral artery ligation.
Figure S4, related to Figure 3. Loss of the NRP1 cytoplasmic tail does not impair VEGF165 binding to NRP1-expressing axons or blood vessels co-expressing NRP1/VEGFR2.
Figure S5, related to Figure 5. VEGFR2 and NRP-1 distribution in wildtype and $Nrp1^{cyto}$ primary arterial endothelial cells and VEGFR2, NRP-1 and EEA1 distribution in wildtype and $Nrp1^{cyto}$ primary arterial endothelial cells 15 minutes following VEGF stimulation.
Figure S6, related to Figure 6. Transferrin trafficking in WT and $Nrp1^{cyto}$ primary arterial endothelial cells.
Figure S7, related to Figure 6. Rescue of impaired VEGFR2 trafficking in $Nrp1^{cyto}$ arterial EC with full length NRP1.
Figure S8, related to Figure 8. Colocalization of PTP1B, VEGFR2 and NRP1 by confocal microscopy.

Supplemental Experimental Procedures

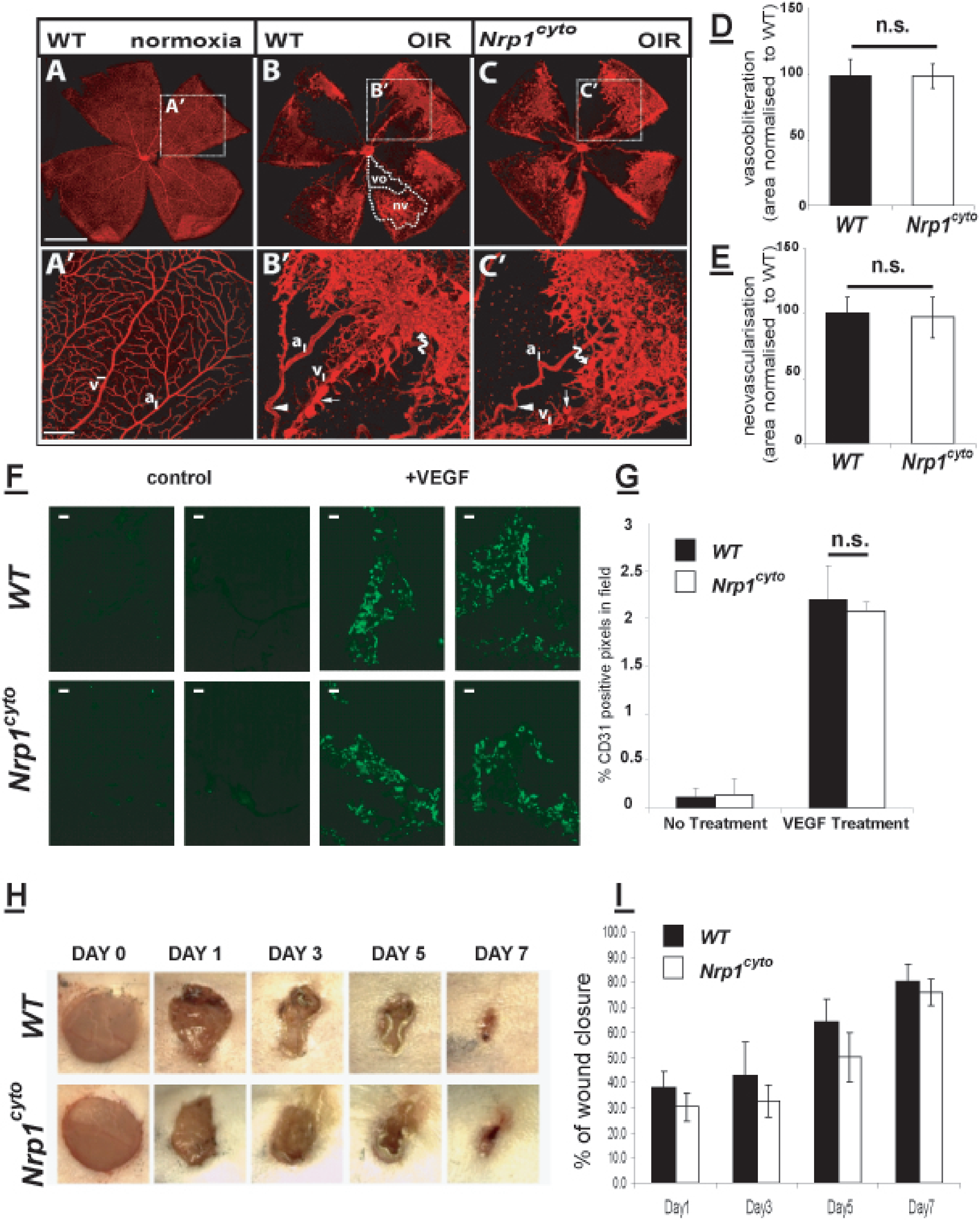

WT normoxia
WT OIR
Nrp1cyto OIR
A
A′
B
B′
C
C′
vo
nv
D
n.s.
vasoobliteration (area normalised to WT)
WT
Nrp1cyto
E
n.s.
neovascularisation (area normalised to WT)
WT
Nrp1cyto
F
control
+VEGF
WT
Nrp1cyto
G
n.s.
WT
Nrp1cyto
% CD31 positive pixels in field
No Treatment
VEGF Treatment
H
DAY 0
DAY 1
DAY 3
DAY 5
DAY 7
WT
Nrp1cyto
I
WT
Nrp1cyto
% of wound closure
Day1
Day3
Day5
Day7

Fig. S1

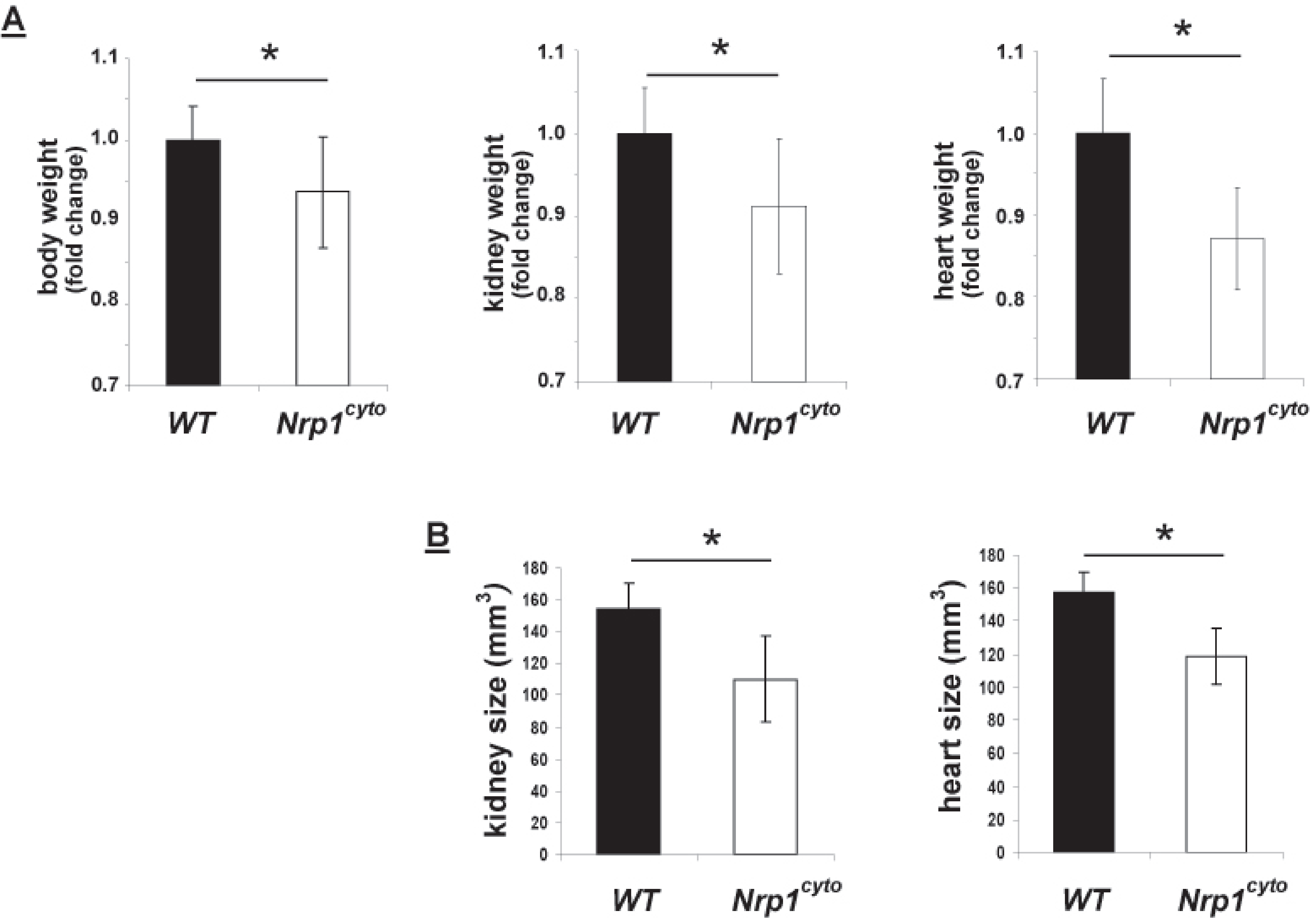
A
body weight (fold change)
kidney weight (fold change)
heart weight (fold change)
WT
Nrp1cyto
B
kidney size (mm3)
heart size (mm3)
*

Fig. S2

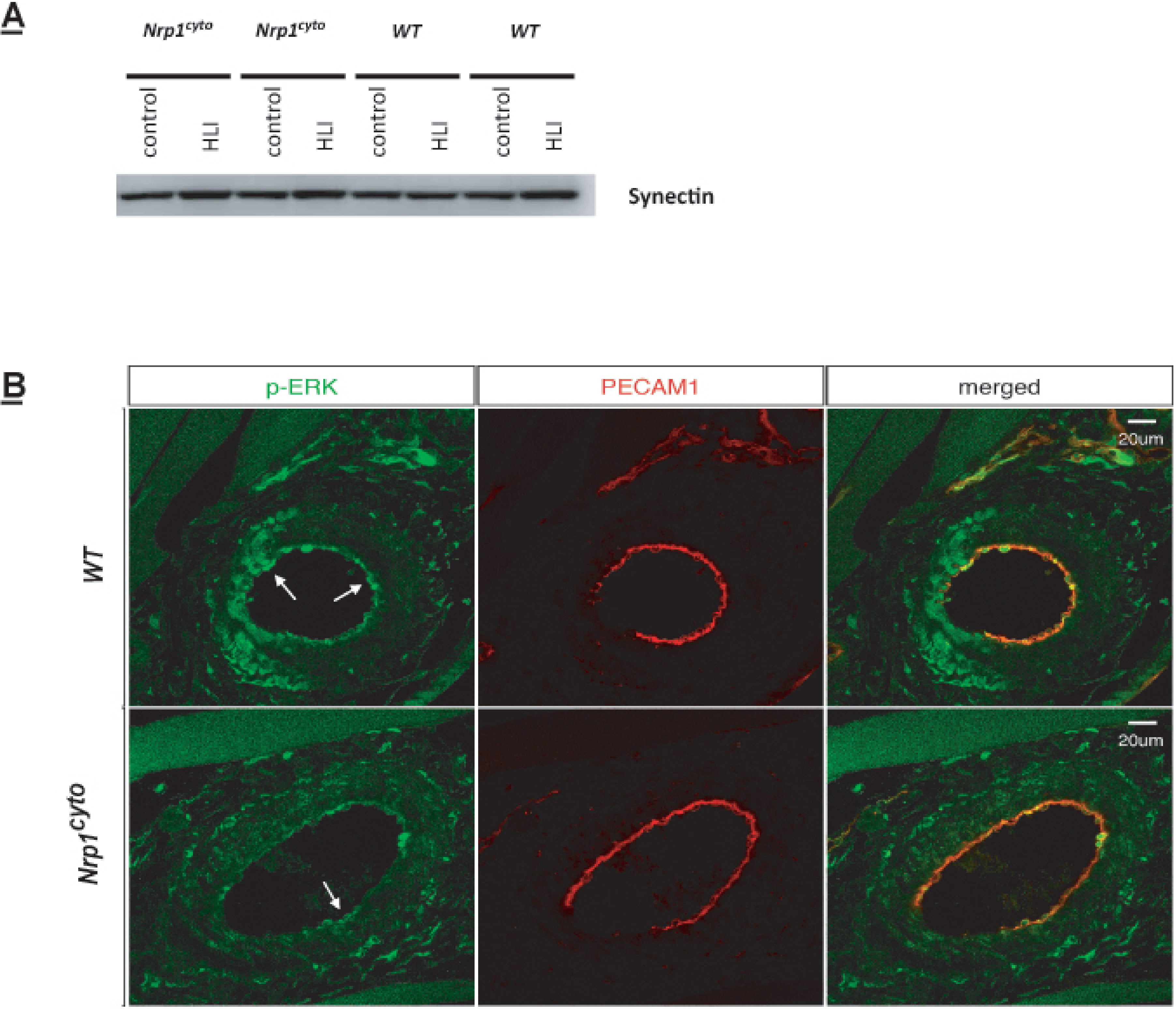
A
Nrp1cyto
Nrp1cyto
WT
WT
control
HLI
control
HLI
control
HLI
control
HLI
Synectin
B
p-ERK
PECAM1
merged
WT
Nrp1cyto
20um
20um

Fig. S3

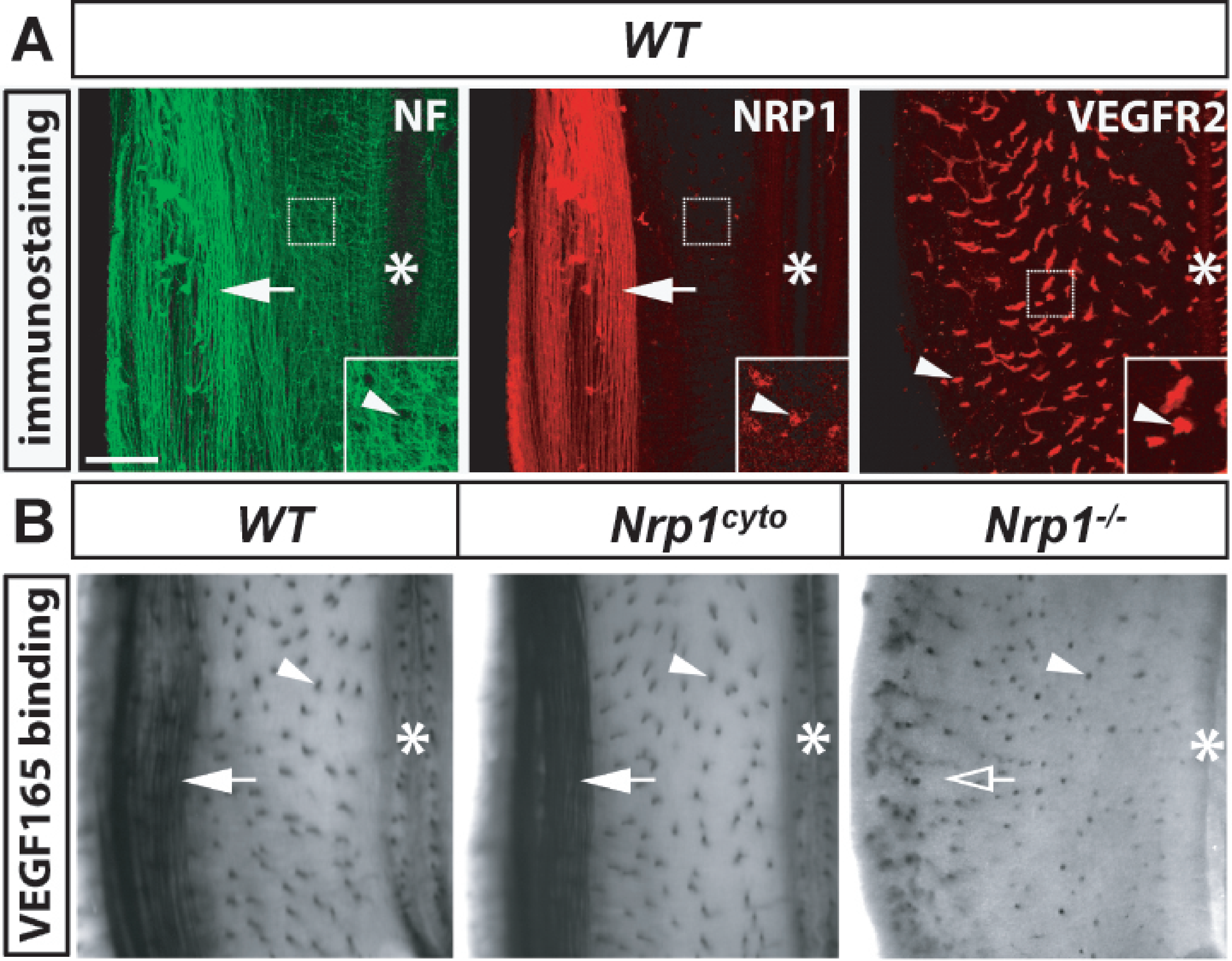
A
WT
immunostaining
NF
NRP1
VEGFR2
B
WT
Nrp1cyto
Nrp1-/-
VEGF165 binding

Fig. S4

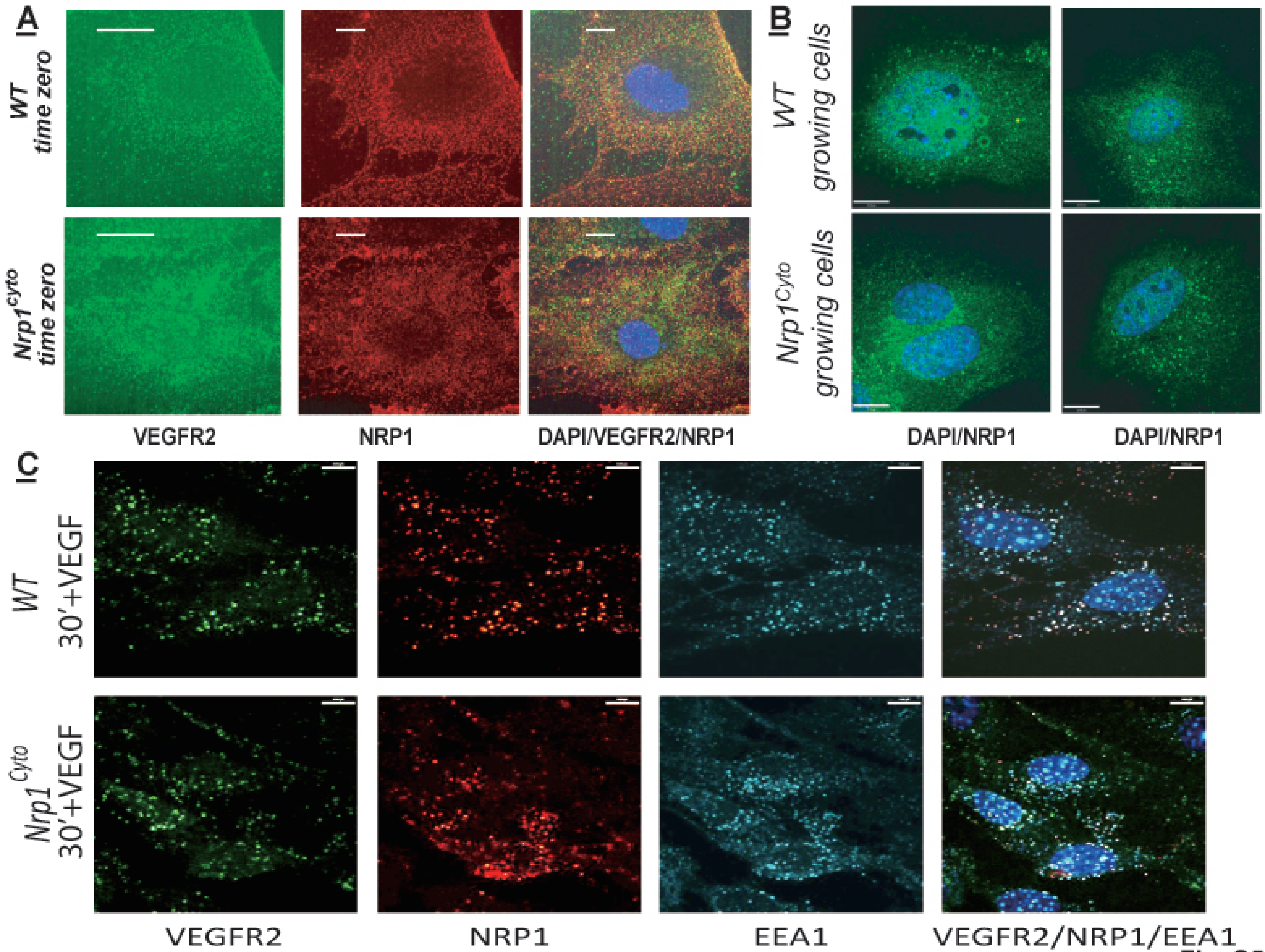
A
WT time zero
Nrp1cyto time zero
VEGFR2
NRP1
DAPI/VEGFR2/NRP1
B
WT growing cells
Nrp1Cyto growing cells
DAPI/NRP1
DAPI/NRP1
C
WT 30'+VEGF
Nrp1Cyto 30'+VEGF
VEGFR2
NRP1
EEA1
VEGFR2/NRP1/EEA1

Fig. S5

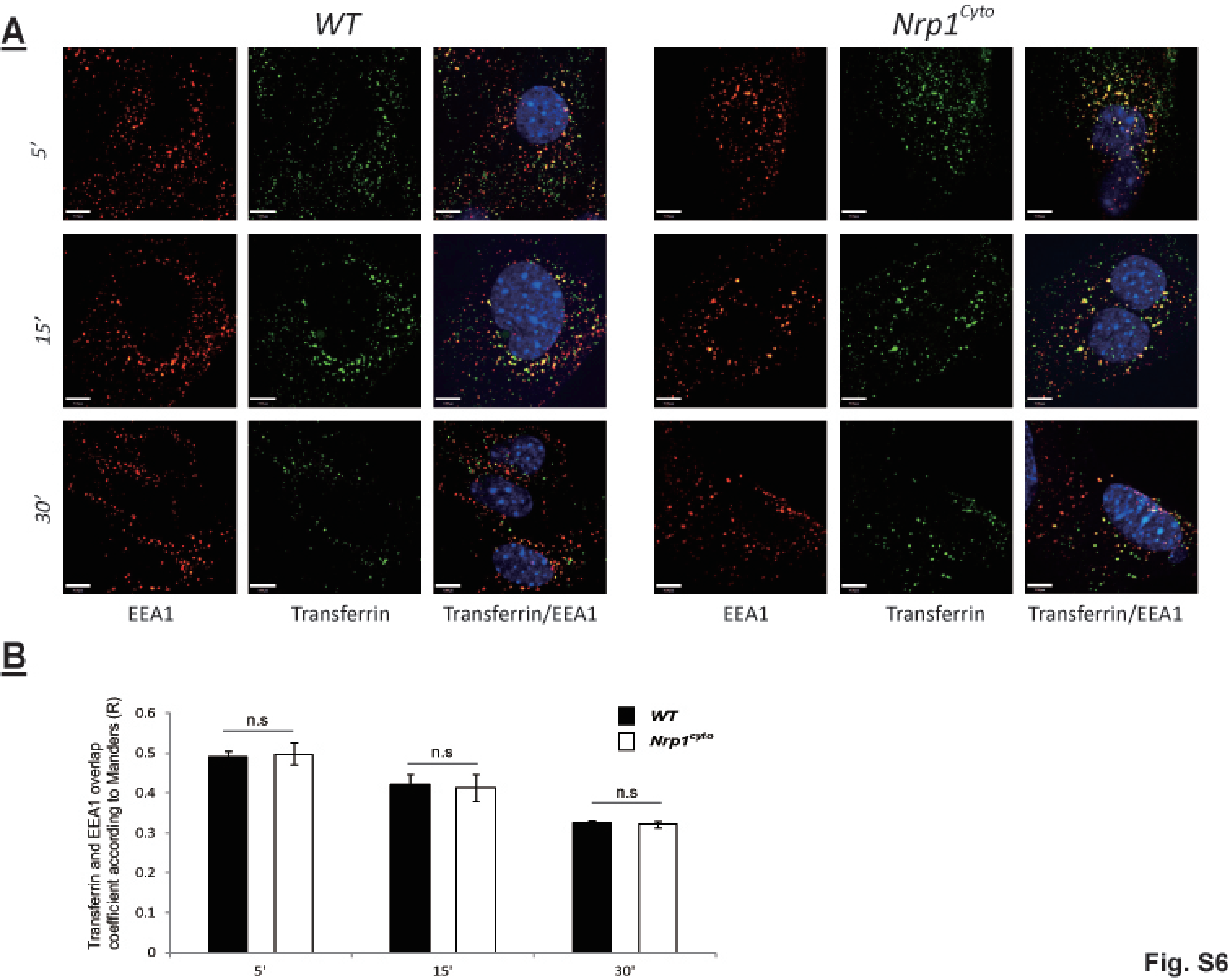
A
WT
Nrp1Cyto
5′
15′
30′
EEA1
Transferrin
Transferrin/EEA1
EEA1
Transferrin
Transferrin/EEA1
B
Transferrin and EEA1 overlap coefficient according to Manders (R)
0.6
0.5
0.4
0.3
0.2
0.1
0
n.s
n.s
n.s
WT
Nrp1cyto
5'
15'
30'

Fig. S6

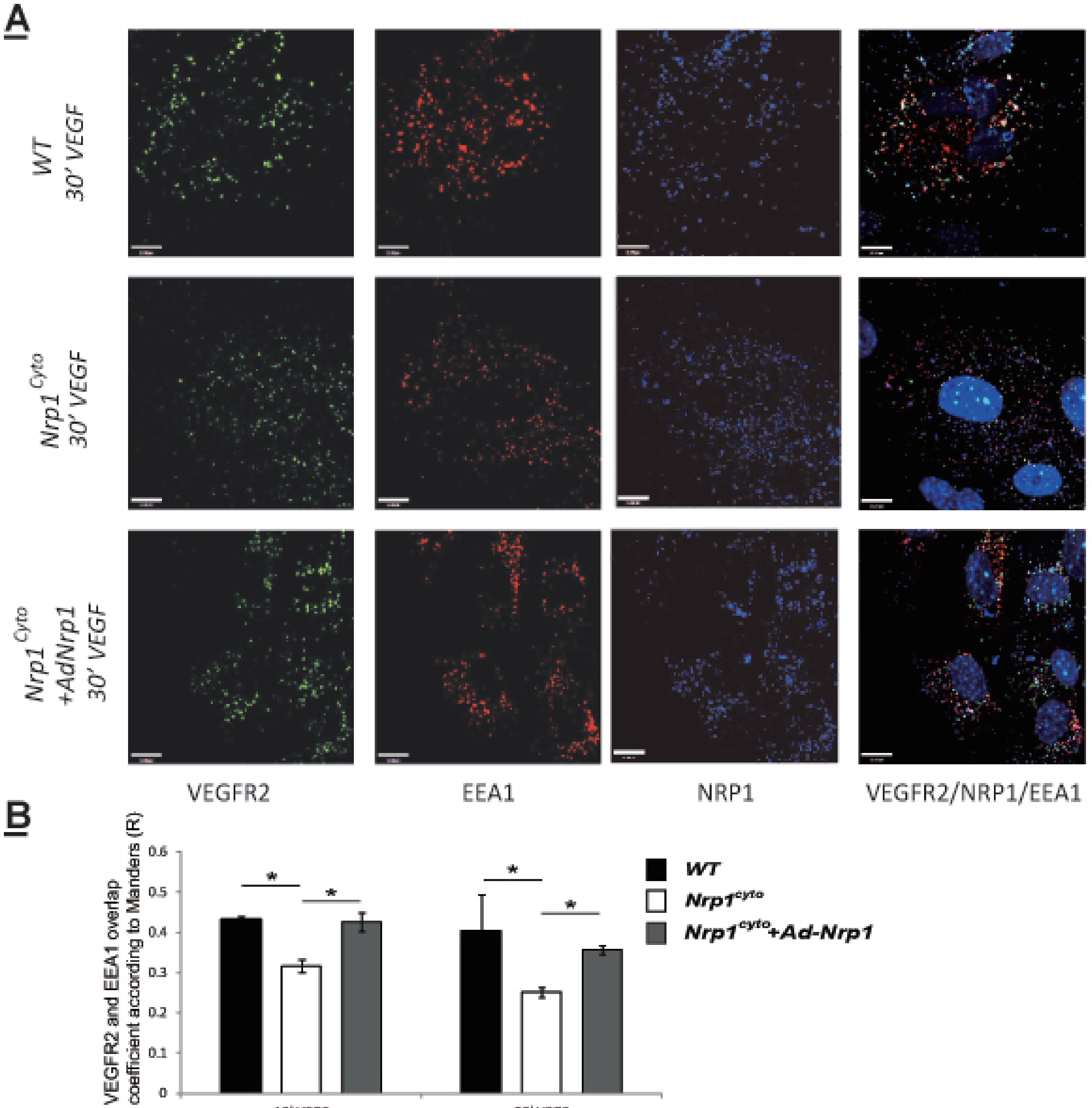

A
WT
30' VEGF
Nrp1 Cyto
30' VEGF
Nrp1 Cyto
+AdNrp1
30' VEGF
VEGFR2
EEA1
NRP1
VEGFR2/NRP1/EEA1
B
VEGFR2 and EEA1 overlap coefficient according to Manders (R)
0
0.1
0.2
0.3
0.4
0.5
0.6
*
*
*
*
15' VEGF
30' VEGF
WT
Nrp1cyto
Nrp1cyto+Ad-Nrp1

Fig. S7

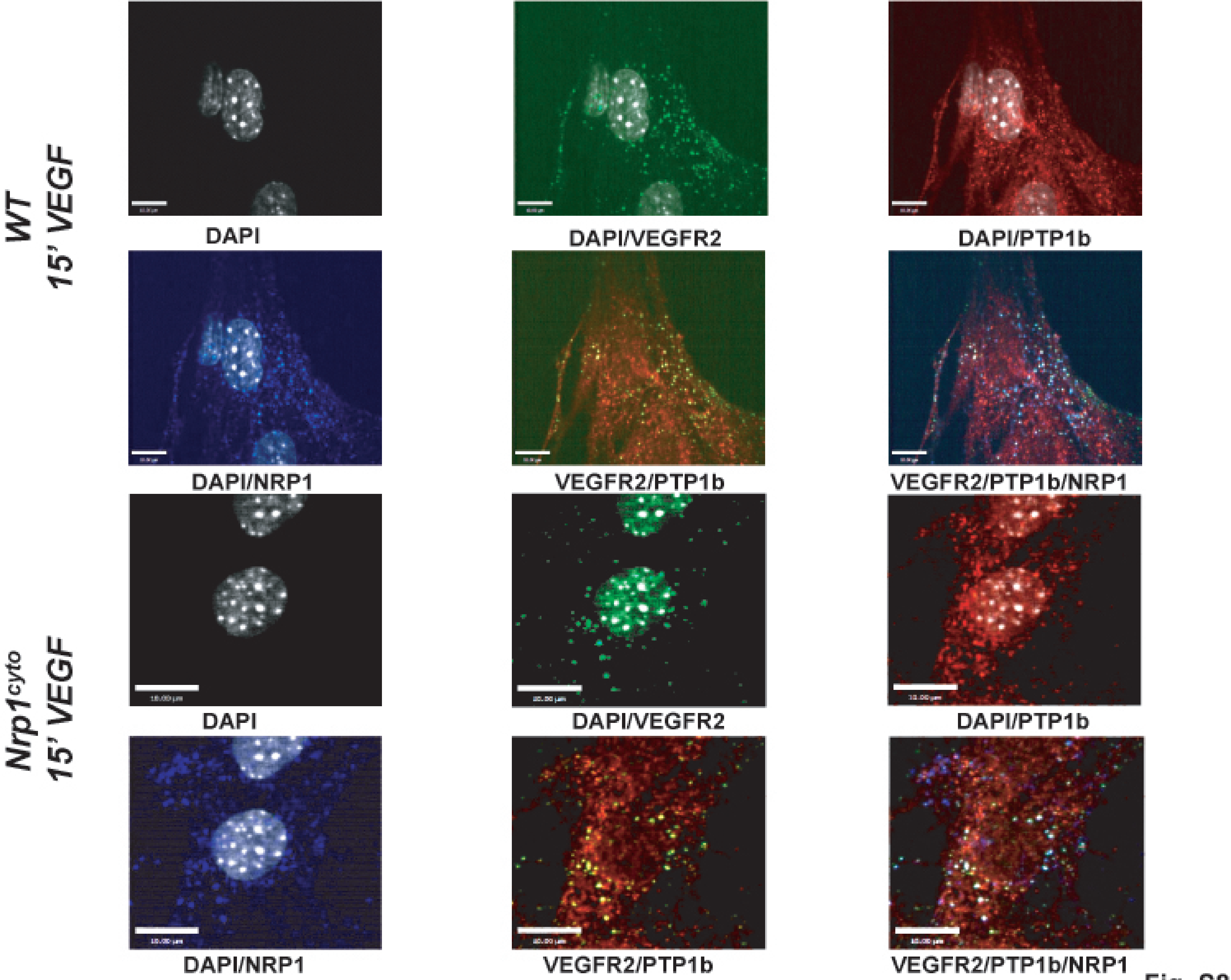
WT
15' VEGF
DAPI
DAPI/VEGFR2
DAPI/PTP1b
DAPI/NRP1
VEGFR2/PTP1b
VEGFR2/PTP1b/NRP1
Nrp1cyto
15' VEGF
DAPI
DAPI/VEGFR2
DAPI/PTP1b
DAPI/NRP1
VEGFR2/PTP1b
VEGFR2/PTP1b/NRP1

Fig. S8

Supplemental Figure 1, related to Figure 1: Loss of the NRP1 cytoplasmic domain does not impair pathological angiogenesis.

(**A-E**) Oxygen-induced retinopathy model. **(A-C)** Isolectin B4 wholemount staining of P17 retinas from wild type and $Nrp1^{cyto}$ littermate mice, subjected to normoxia **(A)** or hyperoxia from P7 to P12 and returned to normoxia from P12 to P17 **(B,C)**; incisions were made in the retina to facilitate flatmounting; the dotted lines in **(B,C)** indicate an example of areas between two arteries that are affected by vasoobliteration (vo) or neovascularisation (nv) emerging from the residual capillary bed. Scale bar: 1 mm. (**A'-C'**) Higher magnifications of areas outlined with a square in **(A-C)**, respectively; owing to hyperproliferation, arteries (a) appear wavy (arrowheads) and veins (v) form vascular tufts (arrows); general vascular overgrowth from the persisting vasculature is indicated with wavy arrows. Scale bar: 200 mm. (**D,E**) Quantitation of the total area of vasoobliteration **(D)** and neovascularisation (mean±SEM, * P > 0.5). **(E)** per retina, normalised to the average of the respective wildtype area in each litter; n$\geq$8 retinas (mean±SEM, * P > 0.5).

(F,G) Angiogenesis in matrigel implants. Matrigel plugs from wildtype and $Nrp1^{cyto}$ mice with and without VEGF-A_{165} treatment were sectioned and immunolabelled for CD31, two representative images per condition are shown in **(F)**, quantification in **(G)**. Scale bar: 70um.

(H,I) Angiogenesis-dependent wound healing model. Wounds were introduced in wildtype and $Nrp1^{cyto}$ mice by punch biopsy and imaged at the indicated times. Representative images of healing wounds are shown in **(H)**, quantification of the area of wound closure in **(I)** (n=5 each, mean$\pm$SD).

Supplemental Figure 2, related to Figure 1: Body weights of wildtype and Nrp1^{cyto} mice, and heart and kidney tissues.

(A) Body weights of wildtype(n=14) and Nrp1^{cyto} (n=23)mice , heart and kidney weights of 8 week female Nrp1^{cyto} (n=9) mice and their wildtype littermates (n=6). (mean$\pm$SD, * $P < 0.05$)

(B) kidney and heart organ size determined by micro-CT. (mean$\pm$SD, * $P < 0.05$)

Supplemental Figure 3, related to Figure 2: Synectin and pERK expression in Nrp1^{cyto} and WT mice following femoral artery ligation.

(A) Western blot analysis of synectin levels in the hindlimb of wildtype and Nrp1^{cyto} mice 3 days following femoral artery ligation.

(B) Sections from mouse hindlimb(thigh) 3 days following femoral artery ligation were immunostained for pERK and PECAM1. Note the elevated pERK lumenal staining(arrows) in the wildtype vessel relative to the vessel from the Nrp1^{cyto} mouse. (scale bar: 20μm)

Supplemental Figure 4, related to Figure 3: Loss of the NRP1 cytoplasmic tail does not impair VEGF165 binding to NRP1-expressing axons or blood vessels co-expressing NRP1/VEGFR2.

(A) Wholemount immunofluorescence staining of E12.5 mouse hindbrain tissue for the axon marker neurofilament (NF, green, left hand panel) and NRP1 (red, middle panel) or VEGFR2 (red, right hand panel); NF and NRP1 staining was performed as a double-labeling of one hindbrain. The insets show higher magnifications of the areas outlined with dotted boxes. Note that NRP1 expression is higher on axons than blood vessels.

(B) Alkaline phosphatase (AP)-coupled $VEGF_{165}$ binding to E12.5 mouse hindbrains of the indicated genotypes. Note that $VEGF_{165}$ binding to axons is preserved in hindbrains lacking the NRP1 cytoplasmic tail, but lost in hindbrains lacking NRP1 completely. Vascular staining is preserved in hindbrains lacking only the NRP1 cytoplasmic tail or lacking NRP1, consistent with expression of VEGFR2. All panels show the pial hindbrain surface; asterisks indicate hindbrain midline and arrowheads examples of radial vessels entering the hindbrain tissue from the perineural vascular plexus; solid arrows indicate longitudinal axons in the dorsal funiculus, and the clear arrow the lack of AP-$VEGF_{165}$ binding to longitudinal axons in the *Nrp1*$^{-/-}$ hindbrain. (scale bar: 200 μm)

Supplemental Figure 5, related to Figure 5: VEGFR2 and NRP-1 distribution in wildtype and Nrp1^{cyto} primary arterial endothelial cells and VEGFR2, NRP-1 and EEA1 distribution in wildtype and Nrp1^{cyto} primary arterial endothelial cells 15 minutes following VEGF stimulation.

(A) Confocal images of VEGFR2 and NRP1 on the cell surface of wild type and Nrp1^{cyto} EC prior to VEGF-A_{165} stimulation shows a similar distribution of VEGFR2 and NRP1 in both genotypes. (scale bar: 9μm)

(B) Confocal images of the intracellular distribution of NRP1 in growing wild type and Nrp1^{cyto} EC shows a similar distribution of VEGFR2 and NRP1 in both genotypes. (scale bar: 13μm)

(C) Confocal images of wildtype and Nrp1^{cyto} primary arterial EC immunolabelled for VEGFR2, NRP1 and EEA1 30 minutes following VEGF-A_{165} stimulation show reduced colocalization of VEGFR2, NRP1 and EEA1 in Nrp1^{cyto} relative to wildtype cells. (scale bar: 9μm)

Supplemental Figure 6, related to Figure 6: Transferrin trafficking in WT and Nrp1^{cyto} primary arterial endothelial cells.

(A) Confocal images of wildtype and Nrp1^{cyto} primary arterial EC immunolabelled for EEA1 following overnight starvation and treatment with transferrin conjugated with Alexa Fluor® 488 show similar transferrin trafficking in Nrp1^{cyto} and wildtype cells. (scale bar: 7μm)

(B) Quantification of EEA1 and transferrin colocalization 5, 15 and 30 minutes following treatment with transferrin (overlap coefficient according to Manders, N=10 independent fields for each time point and cell type, mean±SEM,* P<0.05).

Supplemental Figure 7, related to Figure 6: Rescue of impaired VEGFR2 trafficking in Nrp1^{cyto} arterial EC with full length NRP1.

Wildtype and Nrp1^{cyto} primary arterial EC were transduced with an adenoviral construct expressing full length NRP1 (*Ad-Nrp1*) for two days, serum-starved and then stimulated for 15 or 30 minutes with 50 ng/ml VEGF-A_{165} before immunolabeling for VEGFR2, NRP1, EEA1. **(A)** Confocal images acquired; scale bar: 9um**. (B)** Quantification shows that the proportion of VEGFR2 in EEA1+ endosomes was significantly higher in Nrp1^{cyto} EC containing *Ad-Nrp1* and similar to control EC (overlap coefficient according to Manders, n=10 independent fields for each time point and cell type, mean±SEM, * P<0.05).

Supplemental Figure 8, related to Figure 8: **Colocalization of PTP1B, VEGFR2 and NRP1 by confocal microscopy.**

Confocal images of wildtype and Nrp1^{cyto} primary arterial EC immunolabelled for VEGFR2, NRP1 and PTP1b 15 minutes following VEGF-A_{165} stimulation show colocalization of VEGFR2, NRP1 and PTP1b (scale bar: 10μm).

Supplemental Experimental Procedures

Isolation of murine arterial endothelial cells.

Primary arterial endothelial cells were isolated from adult mouse dorsal aorta as previously described (Chittenden et al., 2006). Briefly, the arteries of 4 mice were harvested and pooled, minced finely with scissors and then digested in 25ml collagenase 0.2% (w/v) at 37°C for 45 min. The crude cell preparation was pelleted and resuspended in DPBS. The cell suspension was incubated with PECAM-1-coated beads (IgG Dynal beads from Dynal Corp., Great Neck, NY) at room temperature for 10 min. with end-over-end rotation. Using a magnetic separator, the bead-bound cells were recovered, washed with DMEM-20%FBS, suspended in 12ml complete culture medium[DMEM containing 20% fetal calf serum, supplemented with 100ug/ml heparin, 100ug/ml ECGF growth supplement (ECGF: Biomedical Technologies, Stoughton, MA), and nonessential amino acids, sodium pyruvate, L-glutamine, and antibiotics at standard concentrations], and then plated in a fibronectin-coated 10cm tissue culture dish. Cells were characterized by staining for the endothelial cell marker PECAM-1 (CD-31) expression by FLOW analysis using FITC anti mouse CD31 and the isotype control FITC Rat IgG2a. 4 wildtype and 4 mutant mice were used for each isolation.

Analysis of VEGFR-2 Internalization by Cell-Surface Biotinylation

Cells were grown to confluence on 10cm fibronectin coated dishes and starved overnight in media with 0.5%FBS. Cells were rinsed 2X with cold PBS, incubated with 5ml/dish EZ-Link Sulfo-NHS-SS-

Biotin(0.25mg/ml) in PBS at +4°C for 30 min. and then quenched by rinsing with cold PBS + 50mM glycine(harvest one plate to determine total biotinylated cell surface protein). Cells were rinsed 1X with cold media + 1% BSA, stimulated with media containing 1% BSA + VEGF-A_{165}(50ng/ml), incubated at 37°C for different times and then rinsed 2X with cold PBS and incubated 2X 20 min. on ice with the membrane-nonpermeable reducing agent GSH(45mM) in 75mM NaCL, 75mM NaOH, 1mM EDTA, 1% BSA. Glutathione was quenched by incubating 2 x 5min. with cold PBS + iodoacetamide(5mg/ml). Cells were rinsed with cold PBS and protein lysates prepared using NP-40 lysis buffer. 200ug of lysate was immunoprecipitated with 50ul of NeutrAvidin beads at +4°C overnight, beads were rinsed 4X with 500ul of NP-40 lysis buffer and resuspended in 60ul 2X Lamelli's SDS-sample buffer. Samples were analyzed by SDS-PAGE followed by Western blotting with anti-VEGFR-2 and anti-NRP1.

Staining of Intracellular Neuropilin1

Cells were plated on gelatin coated glass bottom dishes. Growing cells were fixed for 10 min. with 4% paraformaldehyde(PFA) and permeabilized with 2%PFA, 0.1% Triton X-100, 0.1% NP-40 for 10 min. prior to overnight incubation with Neuropilin1 primary antibody and then secondary antibody conjugated with anti-goat alexa488. Samples were mounted with ProLong Gold DAPI (Invitrogen) and imaged with a Perkin Elmer UltraVIEW VoX spinning disc confocal microscope.

Hindlimb Ischemia Model

As previously described (Chittenden et al., 2006), the femoral artery was ligated at 2 positions spaced 5 mm apart, one just proximal to the caudal femoral artery and the second distal to it and proximal to the popliteal artery. The arterial segment between the ligatures was excised. Tissue perfusion was assessed pre-operatively, immediately post-operatively, and 3, 7, and 14 days after surgical intervention. Flow images of the foot were acquired using a Moor Infrared

Laser Doppler Imager (LDI; Moor Instruments Ltd) at 37.4°C to 38.0°C under ketamine/xylazine (80/5 mg/kg) anesthesia. Data were analyzed with Moor LDI image processing software V3.09 and reported as the ratio of flow in the right/left (R/L) hindlimb after background subtraction. 8 wildtype and 8 mutant mice were used for the experiment and subsequent mCT analysis.

Micro-CT Angiography

2D mCT scans were acquired with a GE eXplore MS Micro-CT System, using a 400 cone-beam with angular increment of 0.5 degrees and 8-27 micron slice thickness at a voltage of 163.2 mAs, 80; kVp. The mCT data were transferred to a Dell Dimension Workstation with real time 3D volume rendering software (version 3.1, Vital Images, Inc. Plymouth, MN) and microview software (version 1.15, GE medical system). Modified NIH ImageJ (National Institutes of Health, Bethesda, MD) and Image Pro Plus (Media Cybernatics) software were used to analyze vessel number, diameter, area, volume, and arterial density. For baseline analysis of heart, hindlimb and kidney in adult, 6 wildtype and 6 mutant mice were used. For analysis of kidney at neonatal day 7, 4 mice of each genotype were used. Micro-CT quantification was done as previously described (Simons, 2008). In brief, the following was done: Data were acquired in an axial mode, covering a volume of 2.0 cm in the *z* direction with a 1.04-cm field of view (covering a single hindlimb). Micro-CT was calibrated using standard wires of different sizes (10, 20, 30, 40, and 50 µm). During postprocessing of the image, a 40 000 gray-scale value was set as a threshold to eliminate bone with minimal sacrifice of vessel visualization. Microview software (GE Healthcare) was used to reconstruct three 2D maximum-intensity projection images (*x*, *y*, and *z* axes) from raw images. A volume of interest was reconstructed of the upper (500 pixels) and lower (250 pixels) hindlimb. Quantification was performed by use of a modified Image Pro-

Plus 5.0 algorithm. The data are expressed as vascular segment number, representing total number of vessels, of specified diameter, counted in 500 *z*sections for thigh or 250 *z* sections for calf images.

Wound Healing Assay and Analysis

Wound healing surgical models were provided by microsurgery core at Yale Cardiovascular Research Center. Briefly, mice were anaesthetized by intraperitoneal injection of a ketamine (100mg/kg)/xylazine (10mg/kg) solution. Full thickness wounds were created with a sterile 6-mm biopsy punch in the back skin (Miltex Inc, PA) without injuring the underlying muscle. Wound regions were digitally photographed using a Leica M125 microscope with a HC80 HD camera (Leica, Germany) on days 0, 1, 3, 5 and 7. Wound area was calculated using NIH ImageJ software. Wound sizes at different time points were expressed as percentage of the wound area on day 0.   6 wildtype and 6 mutant mice were used for the analysis.

In vivo Matrigel Assay and Analysis

C57BL/6 mice were injected subcutaneously with 0.5 ml of growth factor-depleted Matrigel plugs containing heparin (10U) with or without VEGF-A_{165} (100 ng/ml). Seven days later, Matrigel plugs were stained with a monoclonal CD31 antibody. CD31-positive vessels were counted in randomly acquired images using ImageJ.   5 wildtype and 5 mutant mice were used for the analysis.

Oxygen-induced retinopathy

Nursing dams and their pups were maintained at 75±3% O_2 in an oxygen supply chamber from postnatal day (P) 7 to P12, returned to room air on P12 and culled at P17; dams were rested in room air for 1 hour each day. Dissected retinas were labelled with isolectin B4 (IB4) followed by

Alexa-conjugated streptavidin (Invitrogen) to visualize blood vessels. Samples were imaged with an SZX16 stereofluorescent microscope (Olympus) equipped with a digital camera (C4742-95-12HR, Hamamatsu). To determine the size of the avascular and neovascular areas, images were converted to a 16-bit greyscale and analysed with ImageJ version 1.44i (NIH, Bethesda, MD).
